# Activated astrocytes enhance the dopaminergic differentiation of stem cells and promote brain repair through bFGF

**DOI:** 10.1038/ncomms6627

**Published:** 2014-12-17

**Authors:** Fan Yang, Yunhui Liu, Jie Tu, Jun Wan, Jie Zhang, Bifeng Wu, Shanping Chen, Jiawei Zhou, Yangling Mu, Liping Wang

**Affiliations:** 1 Shenzhen Key Lab of Neuropsychiatric Modulation, CAS Center for Excellence in Brain Science, Shenzhen Institutes of Advanced Technology, Chinese Academy of Sciences, Shenzhen 518055, China; 2 State Key Laboratory of Neuroscience, CAS Center for Excellence in Brain Science, Institute of Neuroscience, Shanghai Institutes for Biological Sciences, Chinese Academy of Sciences, Shanghai 200031, China

## Abstract

Astrocytes provide neuroprotective effects against degeneration of dopaminergic (DA) neurons and play a fundamental role in DA differentiation of neural stem cells. Here we show that light illumination of astrocytes expressing engineered channelrhodopsin variant (ChETA) can remarkably enhance the release of basic fibroblast growth factor (bFGF) and significantly promote the DA differentiation of human embryonic stem cells (hESCs) *in vitro*. Light activation of transplanted astrocytes in the substantia nigra (SN) also upregulates bFGF levels *in vivo* and promotes the regenerative effects of co-transplanted stem cells. Importantly, upregulation of bFGF levels, by specific light activation of endogenous astrocytes in the SN, enhances the DA differentiation of transplanted stem cells and promotes brain repair in a mouse model of Parkinson’s disease (PD). Our study indicates that astrocyte-derived bFGF is required for regulation of DA differentiation of the stem cells and may provide a strategy targeting astrocytes for treatment of PD.

## Abstract (web summary)

In the adult brain, the neurotrophic factor basic fibroblast growth factor (bFGF) is predominantly secreted by astrocytes. Here the authors use optogenetics to activate astrocytes, leading to release of bFGF, inducing enhanced dopaminergic differentiation of neural progenitors and promoting brain repair in a mouse Parkinson’s disease model.

## Graphical abstract

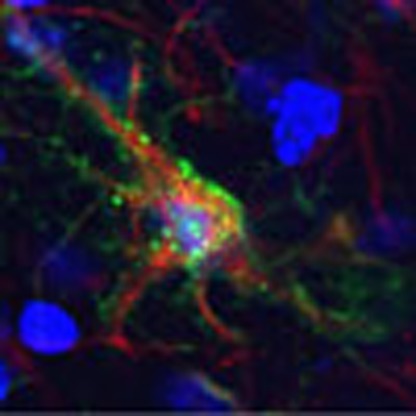

Degeneration and dysfunction of dopaminergic (DA) neurons are involved in various neurodegenerative and mental disorders, such as Parkinson’s disease (PD) and schizophrenia, for which effective therapeutic approaches are still being explored. Direct differentiation of embryonic stem cells (ESCs) intoDA neurons has been attained^1^,^2^ and may provide a source of cell transplantation therapy for PD treatment^3^. A major challenge in enhancing the therapeutic efficacy of ESCs is to promote their proper differentiation and long-term survival in the brain regions that are vulnerable to neurodegeneration in PD.

It has been shown that a number of important molecular pathways play key roles in DA neurogenesis, such as the sonic hedgehog (Shh) signalling^4^,^5^, Wnt/Catenin signalling^6^,^7^,^8^ and FGF signalling pathways^9^,^10^, which regulate the induction, differentiation and maturation of DA neurons. Basic fibroblast growth factor (bFGF), as a physiologically relevant neurotrophic factor, plays an essential role in embryonic development and neural lineage specification of ESCs^11^,^12^. It is one of the crucial elements specifying DA differentiation of ESCs that is widely used to induce the tyrosine hydroxylase (TH)-producing DA neurons^13^,^14^. Fibroblast growth factor receptors (FGFRs) have also been shown to cooperatively regulate the self-renewal and DA differentiation of neural progenitors in the developing midbrain^15^. In PD, a profound depletion of bFGF is found in DA neurons of the substantia nigra (SN)^16^,^17^, whereas co-transplantation of bFGF-producing cells with DA neurons significantly enhances the graft survival and functional recovery^18^,^19^. Together, these findings support the notion that control of bFGF signalling may provide a useful means for optimizing ESC-based therapies for PD.

bFGF expression has been localized to both DA neurons and glial cells^20^. In the adult brain, bFGF is predominantly synthesized and secreted by astrocytes^21^. Nevertheless, it remains unclear whether astrocyte-derived bFGF is sufficient to induce DA differentiation of stem cells and thereby enhance brain repair in a PD model. Emerging studies have demonstrated that astrocytes play important roles in midbrain DA neuron development and modulation of adult neurogenic potential of neuroprogenitors^22^,^23^,^24^. Astrocytes could also secret trophic factors or signalling molecules to protect DA neurons from toxicity in a PD model^6^,^25^,^26^,^27^,^28^,^29^,^30^. The activation of astrocytes could suppress neuroinflammation and improve the resistance of DA neurons^31^,^32^. To enhance the DA differentiation of human EScs (hESCs), researchers have used midbrain astrocytes to substantially potentiate the DA differentiation of the hEScs, and the obtained DA implant yielded a significant restoration of motor function in the parkinsonian rats^33^, suggesting that the midbrain-specific astrocytes play an important role in promoting the differentiation of hESCs and functional recovery in the PD model. Here,we hypothesized that specific activation of the midbrain astrocyte population may increase the synthesis or release of bFGF, which may play a role in promoting the DA differentiation of transplanted stem cells and protecting the residual DA neurons in the PD model.

To test our hypothesis, we used an optogenetic tool to investigate the effects of specific activation of individual astrocyte populations on the DA differentiation of stem cells and the underlying mechanisms. We found that the light-activated astrocytes upregulated the synthesis of bFGF in a tissue-specific manner and significantly promoted the DA differentiation of the hESCs. Most importantly, to our knowledge, we are the first to demonstrate that specific activation of endogenous astrocytes in the SN through an optogenetic approach promoted the astrocyte-specific bFGF release *in situ*, which substantially enhanced the DA differentiation of transplantedstem cells and promoted the regenerative effects in a mouse model of PD.

## Results

### Activated astrocytes induced the differentiation of hESCs

**Figure 1:**
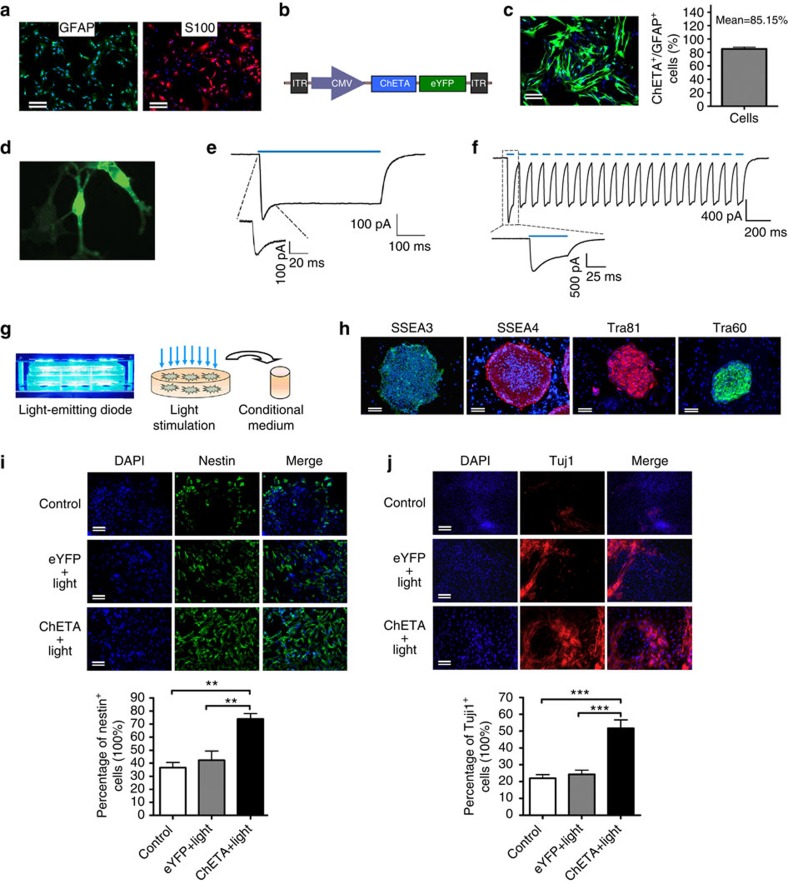
Light stimulation of astrocytes induced the neural differentiation of human embryonic stem cells. (**a**) Immunofluorescence of glial fibrillary acidic protein (GFAP) and S100 on cultured rat-brain-derived astrocytes. Bar, 50 μm. (**b**) Schematic drawing of the *CMV-ChETA-eYFP* lentiviral vector construct. (**c**) Positive *ChETA* expression (green) was observed in the astrocytes transfected with *CMV-ChETA-eYFP* lentivirus (left panel). The percentage of ChETA-expressing astrocytes over the total GFAP-positive cells (right panel); mean±s.e.m. Bar, 25 μm. (**d**) Whole-cell patchclamp was used to record light-evoked photo currents from *ChETA*-expressing astrocytes. (**e**) Light stimulation (500 ms) induced depolarization and a recovery current. (**f**) *ChETA* channel current spike trains induced by light pulses (50 ms, 10 Hz). (**g**) The constructed blue-light- emitting diode was used to illuminate the cultured astrocytes and the conditioned medium was collected after the light stimulation. (**h**) Immunofluorescence of SSEA3, SSEA4, Tra81 and Tra60 on the human embryonic stem cells (hESCs); Bars, 50 μm. (**i**) Immunostaining of Nestin in Control, eYFP+light and ChETA+light groups. The percentage of Nestin-positive cells in the ChETA+light group increased compared with the Control and eYFP+light groups. Bars, 50 μm, (mean±s.e.m., *n*=10). (**j**) Immunostaining of Tuj1 in the Control, eYFP+light and ChETA+light groups. The percentage of Tuj1-positive cells in the ChETA+light group increased compared with the Control and eYFP+light groups. Bar, 50 μm, (mean±s.e.m., *n*=10). All analyses based on Student’s *t*-test, **P<0.01; ***P<0.001. The experiments were repeated three times with similar results.

To determine whether optogenetic manipulation of astrocytes could influence the neural differentiation of the human ESCs, we transfected the rat-brain-derived astrocytes (Fig. 1a) using a lentivirus carrying the *CMV-ChETA-eYFP* construct (Fig. 1b). At 48 h after the transfection, about 85.15% of the GFAP-positive astrocytes were successfully transfected to express *ChETA-eYFP* (Fig. 1c). We then used patch-clamp techniques to investigate the function of ChETA in the transfected astrocytes (Fig. 1d). Stimulating ChETA-expressing astrocytes with blue light (450–490 nm) for 500 ms induced depolarizing currents (Fig. 1e) with a peak amplitude at 142.9±37.7 pA and a steady–state amplitude at 71.2±20.7 pA (*n*=6, Supplementary Fig. 1b). We used 50 ms of light stimuli with a time interval of 50 ms to illuminate the astrocytes, and found that *ChETA* spike trains could be successfully induced by light stimulation (Fig. 1f), suggesting that the protocol we used was sufficient to induce the membrane depolarization of the ChETA-expressing astrocytes.

Then we constructed a blue-light-emitting diode (LED) to illuminate the transfected astrocytes for 1 h (10 Hz and 50-ms stimulus intervals), and the conditioned medium (CM) was collected 24 h after the light stimulation (Fig. 1g). To investigate the effects of CM on neural differentiation of human ESCs, we used human ESC line 9, which expressed pluripotency markers including SSEA3, SSEA4, Tra60 and Tra81 (Fig. 1h). On stimulation with CM from optogenetic-activated astrocytes and other differentiation components, the hESCs gradually differentiated into cobblestone-like cells and then spindled neural progenitors (Fig. 1i,j). Nestin and Tuj1 immunofluorescence staining was performed to evaluate the differentiation efficiency. There were significant increases in the numbers of nestin-positive and Tuj1-positive cells in the ChETA+light group compared with Control and eYFP+light groups (Fig. 1i,j). We also studied the effect of CM on the proliferative potential of hESCs:the 5-ethynyl-2'-deoxyuridine (EdU) incorporation assay showed that the percentage of EdU-positive cells in the ChETA+light group increased significantly compared with Control group (Supplementary Fig. 2d). A co-culture system was set up to study the effect of optogenetic-activated astrocytes on the stem cell differentiation process:the astrocytes expressing ChR2 or eYFP were co-cultured with stem cells and stimulated with blue light (Supplementary Fig. 2a). We observed that the percentage of nestin-positive and Tuj1-positive cells in ChETA+light group increased significantly compared with Control and eYFP+light groups (Supplementary Fig. 2b,c), further supporting the suggestion that optogenetic-activated astrocytes could enhance the neural differentiation of human ESCs.

**Figure 2:**
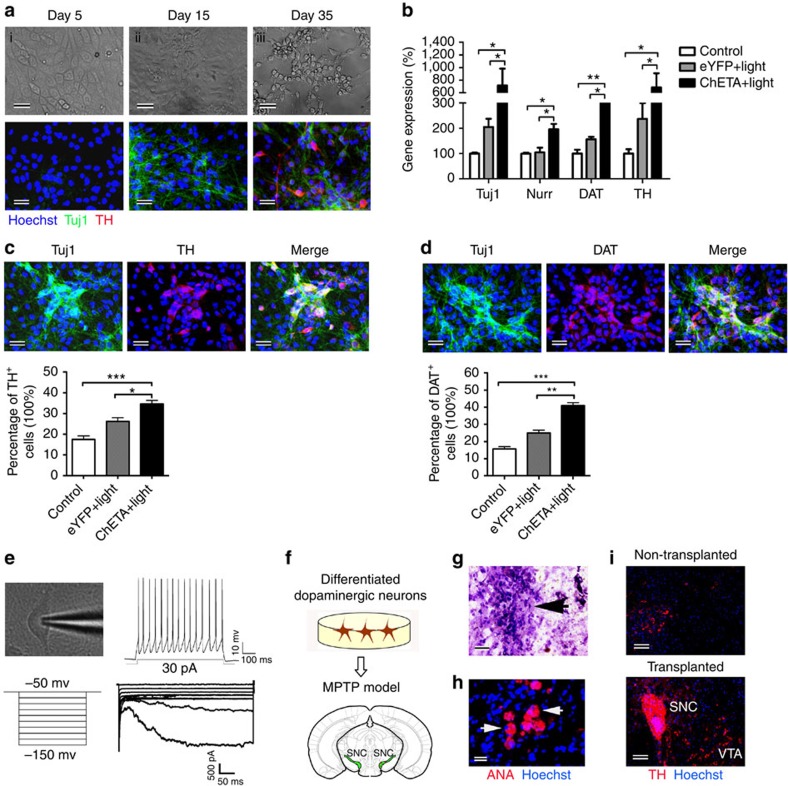
ChETA expression and light stimulation of astrocytes induced the dopaminergic differentiation of human embryonic stem cells. (**a**) hESCs differentiated gradually from cobblestone-like cells (I) into spindled neural progenitors (II) and then typical dopaminergic neurons (III) under the stimulation of conditioned medium from astrocytes (top panel). The dual staining of Tuj1 and TH revealed the progressively differentiated dopaminergic neurons (bottom panel). Bars, 20 μm. (**b**) qRT-PCR analysis showed that gene expression of *Tuj1*, *Nurr*, *Dat* and *TH* increased significantly in the ChETA+light group compared with the Control and eYFP+light groups (mean±s.e.m., *n*=3). (**c**) Immunostaining of tyrosine hydroxylase (TH) in differentiated dopaminergic neurons. The percentage of TH-positive cells in the ChETA+light group increased compared with the Control and eYFP+light groups (mean±s.e.m., *n*=8). Bars, 20 μm. (**d**) Immunostaining of dopamine transporter (DAT) in differentiated dopaminergic neurons. The percentage of DAT-positive cells in the ChETA+light group increased compared with the Control and eYFP+light groups (mean±s.e.m., *n*=8). Bars, 20 μm. (**e**) Whole-cell patchclamp was used to record action potentials from differentiated dopaminergic neurons.The injection of depolarizing currents (30 pA) to dopaminergic neurons led to the firing of action potentials under current-clamp conditions (top panel). The hyperpolarization-activated inward current (*I*_h_) was recorded from the dopaminergic neurons during the hyperpolarization steps (bottom panel). (**f**) Schematic flowchart of the transplantation of the differentiated dopaminergic neurons. (**g**) Representative histological analysis (HE staining) of substantia nigra (SN) at 4 weeks after the transplantation. Bars, 50 μm. (**h**) Anti-nuclear antibody (ANA) staining reveals the origin of the transplanted dopaminergic neurons. Bars, 20 μm. (**i**) Immunostaining of TH of the transplanted and non-transplanted side of substantia nigra. Strong signal should be detected on the transplant (AP=−3.28, ML=−1.5, DV=4.2). Bars, 50 μm. All analyses based on Student’s *t*-test. *P<0.05; **P<0.01; ***P<0.001. The experiments were repeated three times with similar results.

We further investigated the effects of CM from optogenetic-activated astrocytes on the DA differentiation of stem cells: the EdU incorporation assay showed that the percentage of EdU-positive cells was increased significantly in the ChETA+light group (Supplementary Fig. 2e), and the typical morphology of DA neurons was observed at the late stage of differentiation (Fig. 2a). The dual staining of Tuj1 and TH at different stages of the differentiation process showed increased positive signals of Tuj1 and TH with time, supporting the progressive differentiation of DA neurons (Fig. 2a). Quantitative real-time PCR analysis showed upregulated gene expression of *Tuj1, Nurr,TH* and *DAT* in the ChETA+light group compared with those in the Control and eYFP+light groups (Fig. 2b), confirming the DA differentiation of stem cells. Strong expression of TH and dopamine transporter (DAT), markers for DA neurons was observed in the differentiated hES cells (Fig. 2c,d). The percent of TH-positive and DAT-positive cells in the ChETA+light group was 34.54±1.77 and 40.97±1.77%, respectively (Fig. 2c,d), which were significantly higher than those in the Control and eYFP+light groups, suggesting the optogenetic-activated astrocytes could significantly enhance the DA differentiation of ESCs. To test the electrophysiological properties of differentiated DA cells, we performed whole-cell patch-clamp recordings (Fig. 2e) and observed that the recorded cells exhibited action potentials on injection of depolarizing currents (Fig. 2e). We also recorded the hyperpolarization-activated inward current (*I*_h_) (Fig. 2e), which is a common feature of DA neurons^34^. To further evaluate the function of the obtained DA neurons, the differentiated DA neurons were transplanted into the SN region of the MPTP mouse model of PD (Fig. 2f). Four weeks after transplantation, H&E staining showed that cell grafts were positioned within the target brain region (Fig. 2g), and their human origin was confirmed by anti-nuclear Antibody (ANA) staining (Fig. 2h), TH immunofluorescent staining revealed that there were few TH signals in the non-transplanted SN region, while strong TH signals were observed in the transplanted SN region (Fig. 2i). Dopamine level in the treatment group increased significantly compared with the MPTP group (Supplementary Fig. 3a), and motor function assays showed that the transplanted mice had an improved forelimb strength (Supplementary Fig. 3b), a shorter time to orient down and descend in the Pole Test (Supplementary Fig. 3c,d), and a longer distance of locomotion and higher velocity in the OpenField test (Supplementary Fig. 3e,f) compared with the MPTP control group. TH staining in the striatum showed that TH-positive signals increased in the transplanted side compared with the non-transplanted side of the striatum (Supplementary Fig. 3g). Taken together, these data consistently demonstrated that the optogenetic-activated astrocytes could enhance the DA differentiation of human ESCs into functional DA neurons, which promoted brain repair in an MPTP model.

### Light stimulation promoted the synthesis of astrocytic bFGF

**Figure 3:**
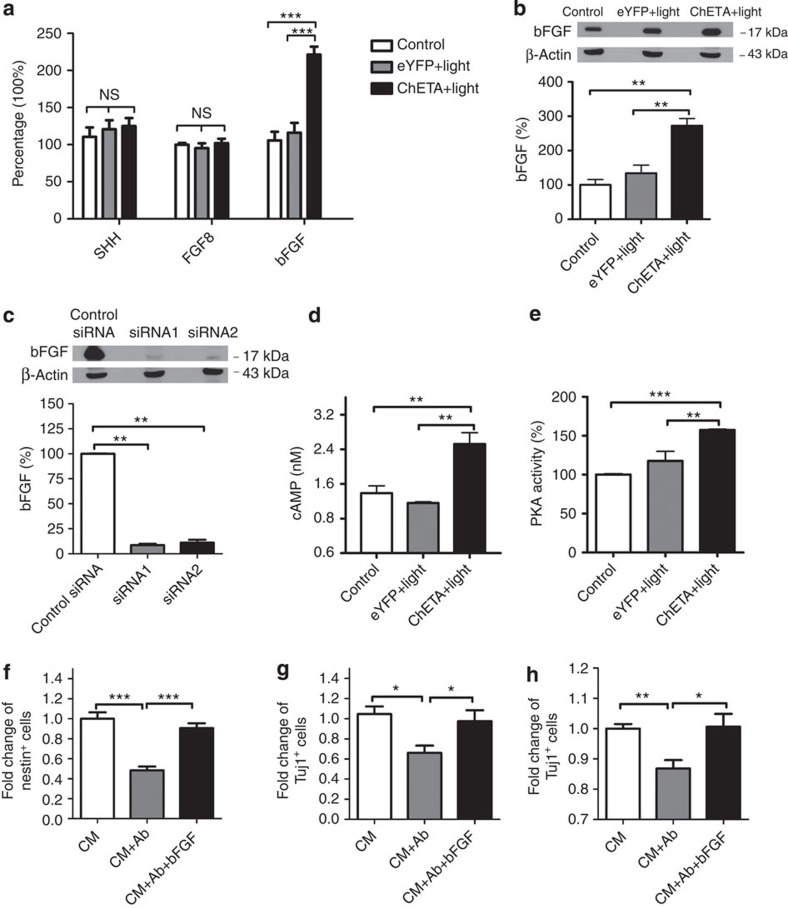
Light stimulation of astrocytes increased the synthesis and release of bFGF. (**a**) Quantification of Sonic hedgehog (SHH), FGF8 and bFGF in the conditioned medium from astrocytes in the Control, eYFP+light and ChETA+light groups (mean±s.e.m., *n*=6). (**b**) Western blot of β-actin and bFGF protein expression in the Control, eYFP+light and ChETA+light groups (upper panel); quantification of bFGF protein expression in the Control, eYFP+light and ChETA+light groups (lowerpanel) (mean±s.e.m., *n*=3). (**c**) Western blot of bFGF and β-actin protein expression in the ChETA+light group treated with two separate bFGF-siRNAs (top panel); quantification of bFGF protein expression in the Control and siRNA-treated groups (bottom panel) (mean±s.e.m., *n*=3). (**d**) Quantification of cAMP in the conditioned medium from astrocytes in the Control, eYFP+light and ChETA+light groups (mean±s.e.m., *n*=5). (**e**) Quantification of Protein Kinase A (PKA) in the conditioned medium from astrocytes in the Control, eYFP+light and ChETA+light groups (mean±s.e.m., *n*=5). (**f**) The fold change of Nestin-positive cells in CM, CM+Ab and CM+Ab+bFGF groups (mean±s.e.m., *n*=8). Ab, anti-bFGF antibody; CM, conditioned medium. (**g**) The fold change of Tuj1-positive cells in CM, CM+Ab and CM+Ab+bFGF groups (mean±s.e.m., *n*=8). (**h**) The fold change of TH-positive cells in CM, CM+Ab and CM+Ab+bFGF groups (mean±s.e.m., *n*=8). All analyses based on Student’s *t*-test. *P<0.05; **P<0.01; ***P<0.001. The experiments were repeated three times with similar results.

SHH, FGF8 and bFGF have beencommonly used to induce the DAprogenitors from human ESCs^33^. We investigated whether ChETA expression and light stimulation could indeed enhance the release of SHH, FGF8 and bFGF from astrocytes: light stimulation did not significantly alter the levels of SHH and FGF8 in CM from astrocytes. However the bFGF level in the ChETA+light group was significantly elevated compared with Control and eYFP+light groups (Fig. 3a). The levels of bFGF in CM increased significantly from 6 to 12 h, followed by a decline at 24 h after the light stimulation (Supplementary Fig. 1c). To exclude the possibility that bFGF elevation is due to cell membrane leakage, we quantified lactic dehydrogenase (LDH) release, and found that there was no significant difference among the three groups (Supplementary Fig. 1d). Next we investigated the biosynthesis of bFGF in astrocytes upon light stimulation. Western blotting showed that the expression level of bFGF was upregulated in the ChETA+light group, and the bFGF expression level in the ChETA+light group was significantly higher than that of Control and eYFP+light groups (Fig. 3b). To verify that light stimulation had indeed upregulated the synthesis of bFGF in ChETA-transfected astrocytes, we performed siRNA knockdown experiments in ChETA-expressing astrocytes. We found that the bFGF expression level after the light stimulation was significantly decreased when the astrocytes were pre-treated with bFGF-siRNAs (siRNA1 and siRNA2), compared with those treated with control siRNAs (Fig. 3c). The level of bFGF-associated signalling molecules, including protein kinase A (PKA) and cyclic AMP (cAMP), was also significantly increased in the ChETA+light group compared with those of the Control and eYFP+light groups (Fig. 3d,e), suggesting that light stimulation activated the PKA/cAMP signalling pathway to promote bFGF synthesis in the transfected astrocytes.

To confirm that bFGF derived from astrocytes^35^ is necessary for the neuronal differentiation of human ESCs, the CM was incubated with an anti-bFGF antibody to neutralize the bFGF (Fig. 3f–h). We found that the bFGF antibody effectively attenuated the neural and DA differentiation capacity of astrocyte-derived CM, indicated by the significantly decreased number of nestin-positive, Tuj1-positive and TH-positive cells in the CM from the CM+Ab group (Fig. 3f–h). Importantly, the decreased neural/DA differentiation capacity of CM could be significantly rescued when exogenous bFGF was added into the previously neutralized CM (Fig. 3f–h). Taken together, these data suggest that bFGF released into astrocytic medium is essential for the neural and DA differentiation of the human ESCs.

### Activated astrocytes enhanced the repair of stem cells

**Figure 4:**
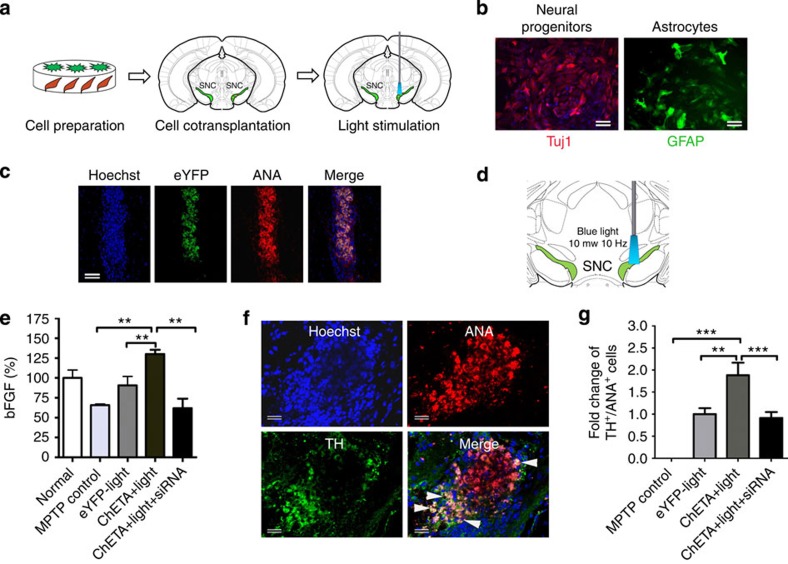
Light stimulation of the transplanted astrocytes enhanced the regenerative capacity of the co-transplanted stem cells. (**a**) Schematic flowchart of the co-transplantation experiment. (**b**) Prepared ChETA-transfected astrocytes (green) and neural progenitors that express Tuj1 (red). Bars, 20 μm. (**c**) Co-localization of the viable transplanted cells using Anti-nuclear Antibody (ANA, red) staining and eYFP fluorescence (green). Bars, 150 μm. (**d**) Schematic drawing of illumination of cells in substantia nigra (SN) region using blue-light-emitting optical fibre. (**e**) Quantification of bFGF level from the SN tissue in the Normal, MPTP Control, eYFP+light, ChETA+light, ChETA+light+siRNAgroups. (mean±s.e.m., *n*=6). (**f**) Immunostaining of TH and ANA revealed the double-positive cells (arrows) among the transplanted stem cells. Bars, 40 μm. (**g**) The fold change of TH^+^/ANA^+^cells in co-transplanted stem cells from the MPTP Control, eYFP+light, ChETA+light and ChETA+light+siRNA groups (mean±s.e.m., *n*=8). All analyses based on Student’s *t*-test. **P<0.01; ***P<0.001. The experiments were repeated three times with similar results.

To investigate whether light stimulation of ChETA-transfected astrocytes could upregulate bFGF level *in vivo* to enhance the differentiation of stem cells, ChETA-transfected astrocytes along with neural progenitors derived from human ESCs were prepared, and co-transplanted into the SN of the MPTP-mouse model of PD (Fig. 4a,b, Supplementary Fig. 4a). One week after the transplantation, the ANA immunofluorescence and eYFP fluorescence confirmed the viability of the co-transplanted cells (Fig. 4c). Then we used a blue-light-emitting optical fibre to illuminate the SN region for 30 min with an average light illumination output power of 10 mW mm^−2^ (Fig. 4d). ELISA analysis showed that 1 week after the light stimulation, the bFGF level in the ChETA+light group increased significantly compared with the Control and eYFP+light groups (Fig. 4e). To verify that the elevated bFGF was mainly derived from co-transplanted astrocytes, we also co-transplanted astrocytes that had been treated with siRNA targeting bFGF; the bFGF level in the ChETA+light+siRNA group decreased significantly compared with the ChETA+light group (Fig. 4e). Then we investigated the change of endogenous DA neurons at each phase after the co-transplantation and light stimulation (Supplementary Fig. 4a). We found that the number of TH-positive cells decreased significantly in MPTP Control, ChETA+light, ChETA+light+SiRNA groups at 7 days after MPTP; however there were more TH-positive cells in the ChETA+light group compared with the other groups after the light stimulation (Supplementary Fig. 4b,c). Light stimulation significantly increased striatal dopamine level (Supplementary Fig. 5a) and improved motor function in the ChETA+light group (Supplementary Fig. 5b–f), including increased forelimb grip strength (Supplementary Fig. 5b), reduced time to orient down and descend (Supplementary Fig. 5c,d), and increased distance of locomotion and average velocity (Supplementary Fig. 5e,f). TH staining in the striatum showed that TH signals increased in the transplanted side of striatum compared with the non-transplanted side (Supplementary Fig. 5g), supporting that light stimulation of co-transplanted cells in sustantianigra promoted functional brain repair in the MPTP model

To evaluate whether elevated astrocytic bFGF influenced the DA differentiation of transplanted stem cells, we performed dual staining of ANA and TH. ANA/TH-positive signals were co-expressed in the transplanted stem cells (Fig. 4f). Quantitative analysis showed that the fold changes of TH^+^/ANA^+^cells in eYFP+light, ChETA+light and ChETA+light+siRNA groups were 1.00±0.14, 1.88±0.28 and 0.91±0.13 respectively, and the number of TH^+^/ANA^+^cells was significantly increased in the ChETA+light group and decreased in the ChETA+light+siRNA group (*P*<0.01) (Fig. 4g).

We also evaluated the effects of intrastriatal co-transplantation of ChETA-transfected astrocytes and ES-derived stem cells in both MPTP and 6-OHDA models after the light stimulation. In the MPTP model, DA differentiation of transplanted stem cells was observed in the striatum (Supplementary Fig. 6a).Dopamine levels in the ChETA+light group were increased significantly compared with the MPTP group (Supplementary Fig. 6b). Motor function assays showed that the co-transplanted mice had an increased forelimb strength (Supplementary Fig. 6c), a shorter time to orient down and descend (Supplementary Fig. 6d,e), a longer distance of locomotion and higher velocity (Supplementary Fig. 6f,g) compared with the MPTP control and eYFP+light groups. In the 6-OHDA model, TH-positive cells were observed among the transplanted stem cells (Supplementary Fig. 7b). The bFGF levels in the ChETA+light group increased significantly (Supplementary Fig. 7c), which was accompanied by elevation of dopamine levels in the ChETA+light group compared with the Control and eYFP+light groups (Supplementary Fig. 7d). Taken together, our data consistently demonstrate that light stimulation specifically upregulated the level of bFGF derived from transplanted astrocytes, enhanced the DA differentiation of stem cells and promoted brain repair in a mouse PD model.

### Activation of endogenous astrocytes enhanced bFGF release

**Figure 5:**
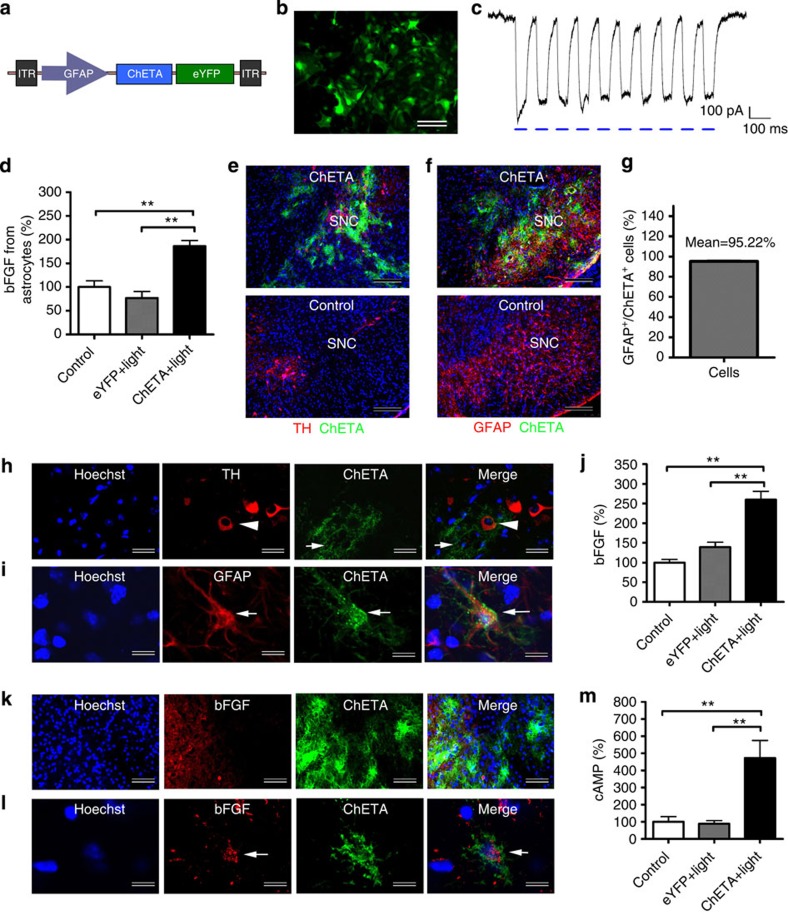
Expression of ChETA in endogenous astrocytes and light stimulation enhanced the bFGF release *in vivo*. (**a**) Schematic drawing of the *GFAP-ChETA-eYFP* lentivirus vector constructs. (**b**) Positive *ChETA* expression (green) was observed in the astrocytes transfected with *GFAP-ChETA-eYFP* lentivirus. (**c**) *ChETA* channel current spike trains induced by light pulses (50 ms, 10 Hz). (**d**) Quantification of bFGF in the conditioned medium from astrocytes in the Control, eYFP+light and ChETA+light groups (mean±s.e.m., *n*=6). (**e**) Immunostaining of tyrosine hydroxylase (TH, red) and *in vivo* expression of ChETA (green) in ChETA group and Control group after injecting the *GFAP-ChETA-eYFP* lentivirus into substantia nigra (SN) region. Bars, 80 μm. (**f**) Immunostaining of GFAP (red) and *in vivo* expression of ChETA (green) in ChETA group and Control group. Bars, 80 μm. (**g**) The percentage of GFAP-positive cells in the total number of ChETA-positive cells, *n*=10. (**h**) High magnification of immunostaining of TH (red, arrowhead) and ChETA (green, arrow) expression in SN. Bars, 20 μm. (**i**) High magnification of immunostaining of GFAP (red) and ChETA (green) expression in transfected astrocytes. Bars, 10 μm. (**j**) Quantification of bFGF from SN tissue in the Control, eYFP+light and ChETA+light groups (mean±s.e.m., *n*=6). (**k**) Immunostaining of bFGF after the light stimulation. The signals (red) were concentrated in the left region occupied by positive ChETA-expressing cells (green). Bars, 50 μm. (**l**) High magnification of immunostaining of bFGF (red) and expression of ChETA (green) in transfected astrocytes. Bars, 10 μm. (**m**) Quantification of cAMP in the SN region in the Control, eYFP+light and ChETA+light groups (mean±s.e.m., *n*=6). All analyses based on Student’s *t*-test. **P<0.01. The experiments were repeated three times with similar results.

Endogenous astrocytes play important roles in regulating the local microenvironmentand influencing the fate of the stem cells^36^. To stimulate the endogenous astrocytes in a cell-type-specific manner, mouse brains were transfected with a construct expressing ChETA-eYFP under the control of *GFAP* promoter to specifically target expression to astrocytes (Fig. 5a). Before the *in vivo* study, expression of ChETA was validated in primary cultured astrocytes. Expression of ChETA was seen 1 week after the transfection (Fig. 5b), and *ChETA* spike trains were successfully induced by light stimulation (Fig. 5c), indicating that there was functional expression of ChETA in astrocytes. Blue light stimulation could also significantly increase the released bFGFfrom ChETA-transfected astrocytes (Fig. 5d). Then we investigated the specific expression pattern of ChETA following injection of the *GFAP-ChETA-eYFP* lentiviral construct into the SN region of the MPTP mouse model. TH staining was performed at 1 week after the injection, and we found that there were few TH-positive signals (red) in the control group: the ChETA signals (green) were mainly concentrated in the SNc region interspersed with residue TH-positive neurons (red) (Fig. 5e). High magnification showed that most of the TH-positive immunosignals were not localized in the ChETA-positive cells (Fig. 5h), indicating that the residual DA neurons in the SN region were not transfected by the *GFAP-ChETA-eYFP* construct. Then we investigated the specific expression of ChETA in endogenous astrocytes through GFAP staining. Strong GFAP-positive signals (red) were observed in the SNc region of the control group (Fig. 5f), and most of the ChETA signals (green)were concentrated in the SNc region where they were co-localized with GFAP fluorescent signals (Fig. 5f,i). Quantification showed that about 95% of the ChETA-positive astrocytes expressed GFAP (Fig. 5g), suggesting that specific ChETA expression in endogenous astrocytes in the SN could be achieved through *in vivo* transfection.

Next we investigated whether light stimulation of endogenous ChETA-transfected astrocytes could promote functional repair in a PD model. First we found that the bFGF level in the ChETA+light group increased significantly compared with the Control and eYFP+light groups (Fig. 5j). To confirm the elevated bFGF expression in the SN, bFGFimmunofluoresence was also performed after the light stimulation. bFGF immunosignals (red) were mainly concentrated in the region containing ChETA-transfected astrocytes (green) (Fig. 5k); whereas few bFGF signals were observed outside the region containing ChETA-expressing cells (Fig. 5k). High magnification showed that the strong positive bFGF signals were detected within the ChETA-positive astrocytes (Fig. 5l). The level of cAMP was also significantly increased in the ChETA+light group compared with that of the Control and eYFP+light groups (Fig. 5m). After the light stimulation, the number of endogenous TH-positive cells in the ChETA+light group increased significantly compared with those of other groups (Supplementary Fig. 8a,b). Dopamine levels in the ChETA+light group also increased significantly compared with the MPTP group (Supplementary Fig. 8c). Motor function assays showed that the treated mice exhibited increased forelimb strength (Supplementary Fig. 8d), a shorter time to orient down and descend in the pole test (Supplementary Fig. 8e,f), and a longer distance of locomotion and higher velocity in the open field test (Supplementary Fig. 8g,h) compared with MPTP control and eYFP+light groups. Taken together, these data demonstrate that the light stimulation specifically activated the endogenous astrocytes *in vivo* and significantly promoted functional brain repair inthe PD model.

### Astrocytes enhanced the DA differentiation and brain repair

**Figure 6:**
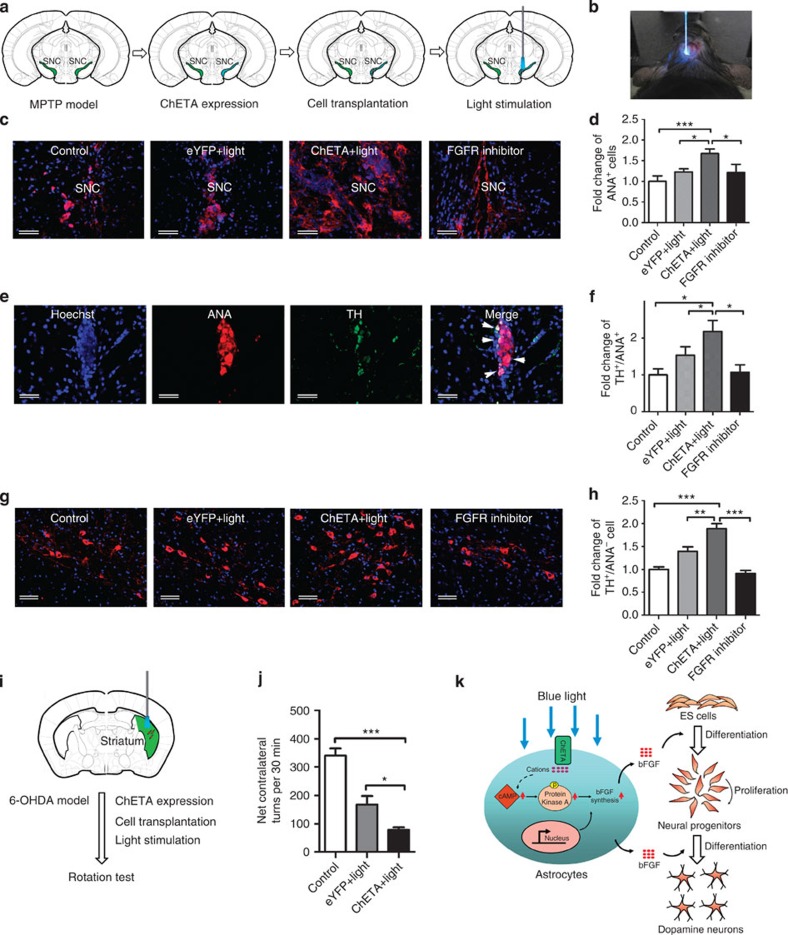
Light stimulation enhanced the dopaminergic differentiation of transplanted cells to promote brain repair. (**a**) Schematic flowchart of the experiment of stem cell transplantation and light stimulation. (**b**) The SN region (AP=−3.28, ML=−1.5, DV=4.3) was illuminated by blue light after the mice were anaesthetized. (**c**) ANA-positive cells were observed in the Control, eYFP+light, ChETA+light and FGFR Inhibitor groups. The signals in the ChETA+light group increased compared with the Control, eYFP+light and FGFR inhibitor groups. Bars, 50 μm. (**d**) The fold change of ANA-positive cells in the Control, eYFP+light, ChETA+light and FGFR Inhibitor groups (mean±s.e.m., *n*=8). (**e**) Immunostaining of TH (green) and ANA (red) of the transplanted stem cells in the SN region. Arrow head, dual stained neurons. Bars, 20 μm. (**f**) The fold change of TH/ANA-positive cells in the Control, eYFP+light, ChETA+light and FGFR Inhibitor groups (mean±s.e.m., *n*=8). (**g**) Immunostaining of endogenous TH-positive cells in the Control, eYFP+light, ChETA+light and FGFR Inhibitor groups. Bars, 50 μm. (**h**) The fold change of endogenous TH-positive cells in the Control, eYFP+light, ChETA+light and FGFR Inhibitor groups (mean±s.e.m., *n*=8). (**i**) Schematic flowchart of the experiment of stem cell transplantation and light stimulation in 6-OHDA murine model. (**j**) Apomorphine-induced net contralateral turns in the Control, eYFP+light and ChETA+light groups. (mean±s.e.m., *n*=5). (**k**) Schematic drawing for the molecular mechanism of the light induced bFGF release to enhance the neural/dopaminergic differentiation of human embryonic stem cells. All analyses based on Student’s *t*-test. *P<0.05; **P<0.01; ***P<0.001. The experiments were repeated three times with similar results.

To investigate the influence of optogenetic-activated astrocytes on the activities of the transplanted stem cells, we expressed ChETA in endogenous astrocytes, transplanted the ES cell-derived neural progenitors and stimulated the SN region using blue light (Fig. 6a,b). Three weeks after the sequential light illumination, the transplanted stem cells (red) were identified with ANA staining, and we found that there were more ANA-positive cells in the ChETA+light group compared with the Control and eYFP+light groups. However, the positive signals were decreased in the FGFR inhibitor SU5402-treated group (Fig. 6c). Quantification showed that the number of ANA-positive cells was significantly increased in the ChETA+light group and decreased in the FGFR inhibitor group (Fig. 6d). To further investigate the differentiation of the transplanted stem cells, TH immunofluorescence and ANA immunofluorescence were recorded simultaneously. We found positive expression of TH among the ANA-positive cells (Fig. 6e). Quantification showed that the numbers of TH^+^/ANA^+^ cells were significantly increased in the ChETA+light group compared with the Control group and decreased in the FGFR inhibitor group (Fig. 6f), suggesting that light stimulation effectively promoted the bFGF dependent-DA differentiation of the transplanted stem cells.

Then we evaluated the activity of endogenous DA neurons after the light stimulation. We found that there were more TH-positive (but ANA-negative) cells in the ChETA+light group compared with the Control and eYFP+light groups; however, the fluorescence intensity decreased in SU5402-treated animals (Fig. 6g). The number of TH-positive cells was significantly increased in the ChETA+light group and decreased in SU5402-treated animals (Fig. 6h), suggesting that light stimulation and stem cell transplantation prevented the decrease of the endogenous DA neurons in the PD model.

The 6-OHDA mouse model of PD (Supplementary Fig. 7a) was used to evaluate functional recovery after optogenetic activation of the astrocytes and stem cell transplantation. Animals received Lentivirus-mediated *in vivo* transfection of ChETA, followed by stem cell transplantation and light stimulation (Fig. 6i). We found that the ChETA+light group showed significantly reduced net contralateral turns in an apomorphine-induced rotational behaviour test compared with Control and eYFP+light groups (Fig. 6j, Supplementary Movie 1), suggesting that ChETA expression and light stimulation were beneficial to the functional improvement in the 6-OHDA-induced PD model. Taken together, our data showed that light-activated endogenous astrocytes could enhance the DA differentiation of transplanted stem cells, increase the number of endogenous TH-positive cells in SN and ameliorate the behaviour deficits in the PD model.

## Discussion

In the current study we investigated the effects of optogenetically activated astrocytes on the DA differentiation of human ESCs and the underlying mechanisms, then we further explored the effects of specific activation of the endogenous astrocytes on the differentiation of transplanted stem cells and functional brain repair. Our study demonstrated, for the first time, that the optogenetically activated astrocytes in the SN upregulated the synthesis and release of bFGF, thereby enhancing the DA differentiation of human ESCs and improving brain repair in a mouse model of PD.

Astrocytes secrete a variety of neurotrophic factors to protect DA neurons and influence the neuronal differentiation of the stem cells. Electrical stimulation of cultured Schwann cells could induce a calcium-dependent release of nerve growth factor, which enhanced the survival of the neurons^37^. Treatment of astrocytes with apomorphine, a potent D1/D2 dopamine receptor agonist, enhanced the biosynthesis of multiple trophic growth factors including brain-derived neurotrophic factor and bFGF^35^, while selective phosphatidylinositol-linked D1-like receptor agonist upregulated the expression level of bFGF in striatal astrocytes in a dopamine D1/D2 receptor-independent manner^21^. To answer the question of whether astrocytes in the SN could be activated to synthesize and release bFGF, we applied optogenetics to regulate the activity of midbrain astrocytes, and found that light stimulation could significantly upregulate the synthesis and release of bFGF. Our co-transplantation assay showed that light stimulation could specifically upregulate the bFGF levels from transplanted astrocytes, demonstrated by the significantly increased bFGF levels in the ChETA+light group and decreased bFGF levels in the ChETA+light+siRNA group (Fig. 4). Using such a cell-type-specific stimulation approach, we further showed that light illumination of endogenous astrocytes could selectively increase the bFGF level in ChETA-transfected endogenous astrocytes (Fig. 5). Taken together, these findings demonstrated that optogenetic activation could specifically upregulate the bFGF level in astrocytes both *in vitro* and *in vivo* (Fig. 6k).

Previous findings have suggested that treatment of astrocytes with D1 receptor agonists stimulated PKA phosphorylation and activated the cAMP/PKA pathway^35^. Optogenetic manipulation of renal tubules could generate cAMP pulses rapidly and reversibly in a cell-type-specific manner^38^. In our study, elevated levels of PKA and cAMP were observed in the light-activated astrocytes, consistent with our findings of upregulated expression of bFGF after the light illumination of astrocytes. Our evidence indicated that light-induced membrane depolarization could activate the cAMP/PKA pathway and increase the cAMP level in both cultured and endogenous astrocytes, thus providing an efficient strategy to directly regulate cAMP level and influence the intracellular signalling pathways in the astrocytes.

The FGF signalling pathway plays diverse roles in embryonic development, cell growth and carcinogenesis^39^. FGF-dependent signalling via phospho-Erk activation was also a major mediator of transitions in the lineage specification of the ESCs^40^, and blocking FGF signalling inhibited the neural induction of human ESCs^12^. Specific and stage-dependent FGF-induced Erk1/2 signalling is also required for the neural specification of the ESCs^41^. Recently, an important study has also shown that increasing FGF signalling in the aged stem cell niche under homeostatic conditions could result in the loss of quiescence of stem cells and diminished regenerative capacity^42^. In our study, we first showed that bFGF derived from the light-activated astrocytes was not only required for the early neural differentiation of the human ESCs, but also needed to enhance the DA differentiation of neural progenitors at the late stage. The co-transplantation assay showed that bFGF derived specifically from transplanted astrocytes significantly enhanced the regenerative effects of stem cells. Last we used optogenetics to specifically regulate the endogenous astrocytes in the SN to promote *in-situ* bFGF release. We found that the elevated bFGF is necessary for enhancing the DA differentiation of the stem cells. This was supported by the findings of the increased expression of DA markers in the ChETA+light group but decreased expression in the presence of FGFR inhibitor. We observed the elevated TH signals in the striatm after intranigral transplantation of differentiated DA neurons or neural progenitors with astrocytes, suggesting that differentiated DA neurons in SN contributed to the increased DA axons and increased dopamine release in the striatum. Using three important approaches we consistently showed that bFGF derived from astrocytes played important and necessary roles to influence the differentiation and proliferation of the stem cells.

Besides the enhancing effects on the neural differentiation of the stem cells, bFGF could also protect nigral DA neurons from neurotoxin-induced cell death^16^. bFGF increased the BrdU-labelled proliferative cells in the SN of MPTP-treated mice^43^. Endogenous bFGF deficiency in FGF-2^−/−^mice caused a significant decrease in the DA neurons after neurotoxin application^44^. Grafts of bFGF-expanded cells contained significantly more TH-positive cells than FGF-8 expanded cells, and induced a better functional recovery in 6-hydroxydopamine-lesioned rats^45^. In our study we observed that the elevated bFGF promoted the proliferation and enhanced the DA differentiation of stem cells. Importantly, we also observed an elevated number of endogenous DA neurons and improved motor functions in the SN after light stimulation of the endogenous astrocytes. Our data suggested that boosting endogenous astrocyte activity through optogenetics could be beneficial to the survival of the remaining nigral DA neurons, elevated dopamine level and the amelioration of the behaviour deficits in the model of PD. It would also bring astrocytes as new therapeutic targets and help to ameliorate the neurodegenerative and behavioural effects in the treatment of PD.

Besides neurotrophic factors such as bFGF, recent studies have also shown that light stimulation could lead to gliotransmitter release from opsin-gene-expressing astrocytes. Optogenetically activated local astrocytes showed increased release of ATP^46^,^47^ and glutamate^48^. Optogenetic activation of astrocytes could also activate the neurons to trigger respiratory responses^49^, modulate motor behaviour^48^, enhance synaptic transmission^50^ and ameliorate ischemic brain damage^51^. On the basis of these studies we further showed that optogenetic activation of astrocytes could enhance the release of bFGF to promote the DA differentiation and functional repair in PD.

In conclusion, our study first discovered that the specific activation of midbrain astrocytes increased the synthesis and release of bFGF, which directed the differentiation of the transplanted hESCs to DA neurons.Using the optogenetic approach to regulate astrocytes in a tissue-specific manner would also be helpful to dissect the elaborate interactions between astrocytes and neurons in PD, which will contribute to the further understanding the biological role of the astrocytes in neural degenerative disease. Furthermore, clarification of the mechanisms of crosstalk between astrocytes, an important hosting cellular component, transplanted hESCs and the endogenous DA neurons will be crucial for an in-depth understanding of the development, survival and functional integration of DA neurons, and may provide the experimental evidence for novel therapeutic approaches for brain diseases involving dysfunction of DA neurons.

## Methods

### *ChETA* expression in astrocytes using lentiviral vectors

Lentiviral vectors carrying the *ChETA-eYFP* or *eYFP* gene (Fig. 1b) were constructed. In brief, the *CMV* or *GFAP* promoter was cloned into the *pLenti-CaMK IIa-ChETA-eYFP* or *pLenti-CaMK IIa-eYFP* plasmid (provided by Dr Karl Deisseroth in Stanford University). Then 293FT cells were transfected with the lentiviral vector pCMVΔR8.74 and pMD2G, and a high titre lentivirus was produced. Twenty-four hours after the transfection, 293FT cells were transferred to serum-free medium containing 5 mM sodium butyrate; the supernatant was collected and concentrated by ultracentrifugation at 50,000 *g*, then phosphate-buffered saline (PBS) was used to re-suspend the viral pellets. Lentivirus was used to express the ChETA fusion protein in the cultured astrocytes. Primary cultured astrocytes were transfected with the Lentivirus (10^9^ TU ml^−1^) in a serum-free medium for 48 h. Expression of ChETA-eYFP in astrocytes was monitored by observation of green fluorescent protein (GFP) expression. To evaluate the transfection efficiency, the astrocytes were counterstained with a GFAP antibody (Abcam). The ChETA-positive cells and GFAP-counterstained cells were counted in 10 high-magnification fields, and the percentage of transfected astrocytes over the GFAP-counterstained astrocytes was calculated.

### *In vitro* stimulation of *ChETA*-expressing astrocytes

The primary rat striatal astrocytes were prepared from the striata of neonatal Sprague–Dawley rats. The neonatal striata were trypsinized for 10 min and tissues were dissociated and plated into six-well plates^21^. The astrocytes were divided into three groups: control group without ChETA expression and light stimulation; eYFP+light group, which expressed eYFP and was stimulated by light; and ChETA+light group, which expressed ChETA and was stimulated with light. A homemade light emitting diode (LED) was used to stimulate the astrocytes for 1 h using blue light with a wavelength of 460–485 nm, and the average output power of the LED was 1.65 mW mm^−2^. At 24 h after the light stimulation, the CM from all the groups was harvested and used for inducing the neural differentiation of ESCs or other analyses.

### *In vitro* culture and DA differentiation of hESCs

The experiments were approved by the Ethics Committee of the Shenzhen Institutes of Advanced Technology, ChineseAcademy of Sciences. The hESCs line H9 was kindly donated by Shenzhen Beike Biotechnology Company, and the culture of hESCs was performed as described previously^33^. In brief, the cells were grown on mouse embryonic fibroblasts and fed every 24 h with Knockout-DMEM (Invitrogen, Carlsbad, CA, USA) supplemented with 20% KO-serum replacement (KO-medium, Invitrogen). The hESCs were passaged every 7 days when the cells reached 80% confluence. At each passage, the cells were treated with collagenase type IV (Invitrogen) for 10 min, and then the cells were gently scraped from the dish and split 1:4 among the coated culture plates.

For the neural differentiation of hESCs, the cells were firstly dissociated to form embryonic bodies and suspended in culture for 4 days. The embryonic bodies were next plated on the dish in a medium containing insulin, transferrin and selenit (ITS medium, R&D Systems, Minneapolis, MN, USA) and cultured for 9 days. Then the cells were trypsinized, transferred to the polyornithine+laminin-coated plates and cultured in DMEM/F12 supplemented with N2 medium (R&D Systems) for 6 days; at this stage, the CM from different groups of activated astrocytes was added into the culture medium to evaluate the effects of CM on the neural differentiation of the hESCs. The DA differentiation of stem cells was further induced by replacing N2 medium with brain-derived neurotrophic factor (25 ng ml^−1^) and 0.5% fetal bovine serum(Invitrogen), and culturing the cells for 10 days. The medium in each group was changed three times per week and phase-contrast images were taken using a microscope (IX71, Olympus, Center Valley, PA, USA).

The cells were divided into three groups: the Control group was cultured in neural differentiation medium only; the eYFP+light group was cultured in half neural differentiation medium and half CM from *eYFP*-transfected astrocytes; the ChETA+light group was cultured in half neural differentiation medium and half CM from *ChETA-eYFP*-transfected astrocytes. The eYFP+light group was included in the assay to exclude non-specific effects of virus expression and light stimulation. The efficiency of neural/DA differentiation was examined by immunostaining using specific antibodies against nestin, Tuj1and tyrosine hydroxylase (TH).

For the co-culture of ES cells and transfected astrocytes, the stem cells were grown on top of a monolayer of astrocytes expressing ChETA-eYFP or eYFP alone (Supplementary Fig. 2a). The cells were divided into the Control group, eYFP+light group and ChETA+light group. The co-cultured cells were stimulated with a homemade LED for 1 h every 3 days using blue light with a wavelength of 460–485 nm. The efficiency of neural/DA differentiation was examined by immunostaining of Nestin/Tuj1 at 15 or 35 days of differentiation.

### Gene expression analysis using RT-PCR

At a late stage of DA differentiation, total RNAs were extracted from cells in Control, eYFP+light and ChETA+light groups using Trizol reagent. RT-PCR was performed using PrimeScript One Step RT-PCR Kit (Takara, Shiga, Japan) and 1 μg of purified RNA. For real-time PCR, analysis was performed in triplicate using LightCycler480(Roche, Switzerland) with Thunderbird SYBR qPCR mix (Toyobo) and primers listed in Supplementary Table 1. Gene expression analysis was performed using the relative cycle threshold method, normalized to GAPDH expression, and the fold changes were calculated relative to the control group.

### *bFGF* knockdown

Two separate standard siRNAs (Invitrogen), S132467 (sense: 5′- ACACUUACCGGUCACGGAATT -3′; antisense: 5′- UUCCGUGACCGGUAAGUGUTG -3′) and S222026 (sense: 5′- GCCUGGAGUCCAAUAACUATT -3′; antisense: 5′- UAGUUAUUGGACUCCAGGCGT -3′) that target the sequences of rat *bFGF* genes (GeneBank accession: NM_019305.2) were used to suppress the bFGF gene expression. The ChETA-transfected cells were divided into three groups: the Control group was treated with negative control siRNA; the siRNA1 group was treated with S132467; and the siRNA2 group was treated with S222026. In brief, the transfection reagents (Invitrogen), 82 μl culture medium without serum and antibiotics and 15 μl of 5 μM siRNA (S132467, S222026 or negative control siRNA, Invitrogen) were mixed together and incubated for 5–10 min at room temperature to allow the formation of transfection complexes, and then the complexes were added drop-wise onto the ChETA-expressing cells, giving a final siRNA concentration of 5 nM. The cells were incubated with the transfection complexes for 48 h under normal growth conditions without changing the medium. Cells in the three groups were stimulated with blue light for 1 hour and the protein was extracted 12 h after the light stimulation for western blot analysis.

### Western blotting

Astrocytes were lysed and the protein was extracted using lysis buffer (NE-PERNuclear and Cytoplasmic Extraction Reagents, Pierce/Thermo Fisher Scientific, Waltham, MA, USA). Total proteins were determined using a Bicinchoninic Acid Protein Assay Kit (Pierce/Thermo Fisher Scientific). The samples were loaded onto 12% SDS–polyacrylamide gel electrophoresis separating gel and run at a voltage of 120 V for 2 h. The total β-actin and bFGF contents were quantitated using mouse anti-β-actin antibodies (1:15,000, Sigma-Aldrich, St Louis, MO, USA) and rabbit anti-bFGF antibodies (1:100, Santa Cruz, Dallas, TX, USA), followed by horseradish peroxidase-conjugated goat anti-rabbit IgG (1: 4,000, KPL, MD, USA). Colour development was achieved by using the ECL Western Blotting Detection Kit (Pierce/Thermo Fisher Scientific). Supplementary Figure 9 shows images of the full uncropped blots.

### Quantitative determination of bFGF, cAMP and PKA

The concentrations of bFGF in cell extracts or tissue homogenate were determined using FGF basic ELISA Kit (Invitrogen). The concentrations of cAMP were determined using Cyclic AMP Direct EIA Kit (Arbor Assays, Ann Arbor, MI, USA). The concentrations of PKA were determined using PKA activity Kit (Arbor Assays). All the experiments were carried out according to the manufacturer’s instructions. The optical density was determined at 450 nm using the microplate reader (Synergy 4, Winooski, VT, USA). The concentrations of bFGF, cAMP and PKA were determined, normalized to the Control group and compared among the different groups.

### Immunostaining

Cultured cells were fixed with 4% paraformaldehyde (PFA), then incubated with mouse antibodies to SSEA3 (1:500, Invitrogen), SSEA4 (1:500, Invitrogen), Tra81 (1:500, Invitrogen), Tra60 (1:500, Invitrogen), Nestin (1:400, Sigma), Tuj1 (1:500, Sigma), TH (1:400, Millipore) or DAT (1:100, Santa Cruz) to characterize the molecular markers. After washing three times, the slides were incubated with FITC- or PE-conjugated secondary antibody (1:500, Invitrogen). Finally the sections were counterstained with 4′,6-diamidino-2-phenylindole (DAPI) or Hoechst 33342 to stain the nuclei and then mounted.The number of Nestin-positive, Tuj1-positive, TH-positive and DAT-positive cells in each group was separately counted in 10 high-magnification fields; the percentage of positive cells was calculated and compared among different groups.

For the brain samples, the brains were fixed in 4% PFA at 4 °C overnight and cryosectioned at a thickness of 20 μm. The sections were then rehydrated and blocked by goat serum. The sections were incubated with primary antibodies to bFGF (1:200, Santa Cruz), nestin (1:400, Abcam, Cambridge, MA, USA), TH (1:500, Millipore, Billerica, MA, USA) or anti-nuclear antibody (ANA, 1:400, Millipore). The sections were then washed and labelled with the fluorescent secondary antibody (1:500, Invitrogen). The sections were counterstained with Hoechst 33342 and then mounted for image acquisition. The images were taken under the microscope (ECLIPSE 50i, Nikon, Melville, NY, USA). The number of TH-positiveor ANA-positive cells in the SN or striatum region was counted in 12 sections (three adjacent levels) from each mouse, and each group contained three mice. The mean cell number per section was normalized to the control group and compared among different groups.

### EdU incorporation analysis for cell proliferation

The 5-ethynyl-2'-deoxyuridine (EdU) incorporation analysis was performed using a Click-iT Alexa Fluor 488 EdUImaging Kit (Invitrogen, Carlsbad, CA, USA). In brief, after 24-h incubation with 10 Mm EdU, the cells were fixed with 4% PFA for 15 min and 0.5% Triton X-100 was used to permeabilize the cells. The cells were incubated with the Click-iT reaction cocktail (Click-iT reaction buffer, CuSO4, Alexa Fluor 488 Azide, and reaction buffer additive) for 30 min while being protected from light. The cells were then incubated with Hoechst 33342 to stain the nuclei, and images were acquired. Eight images from each group were selected; the EdU-positive cells were counted using Image-Pro Plus software (Media Cybernetics, Rockville, MD, USA). The experiments were repeated three times for accuracy and the differences among the groups were compared.

### Establishment of the MPTP/6-OHDA model and cell transplantation

All animal experimental protocols were approved by the Research Committee of the Shenzhen Institutes of Advanced Technology, Chinese Academy of Sciences. One hundred and ninety male C57 mice (6–8 weeks old) were used in the study. To establish the MPTP model of PD, C57 mice were given four intraperitoneal injections of 1-methyl-4-phenyl-1,2,3,6-tetrahydropyridine (MPTP, Sigma) at 2-h intervals, with a total dose per mouse of 100 mg kg^−1^. 6-hydroxydopamine (6-OH-DA)-induced murine PD model was established as described previously^52^. One week after establishing the MPTP model, the mice were anaesthetized with an intraperitoneal injection of phenobarbitol sodium (100 mg kg^−1^). The head skin was cleaned with a 70% isopropanol solution and an incision was made along the midline. For the transplantation of differentiated DA neurons, the mice were divided into three groups: the Normal, MPTP Control and Treatment groups: the DA neurons (2 × 10^5^) were injected into the SN region (AP=−3.28, ML=−1.5, DV=4.3, ALLEN Mouse Brain Atlas) of Treatment groups. For the cell co-transplantation experiment, the mice were divided into three groups: the Control, eYFP+light and ChETA+light groups: the treated ChETA-transfected or eYFP-transfected astrocytes (1 × 10^5^) and human ESCs-derived neural progenitors(1 × 10^5^) were mixed in 5 μl DMEM medium and injected to the SN region (AP=−3.28, ML=−1.5, DV=4.3) or striatum region (AP=0.5, ML=−2.0, DV=4.3) of ChETA+light group or eYFP+light group. For the stem cell implantation after the activation of endogenous astrocytes, the mice were divided into three groups: the Control, eYFP+light and ChETA+light groups: hESC-derived neural progenitors (2 × 10^5^) were injected into the SN region (AP=−3.28, ML=−1.5, DV=4.3) of eYFP+light group and ChETA+light group. Experimenters were blinded to the treatment groups, sample sizes were based on previous experiments. Four mice were excluded from the analysis because of problems with the injection.

### *In vivo* transfection of endogenous astrocytes and light stimulation

One week after establishing the MPTP mouse model, 3 μl lentivirus solution (10^9^ TU ml^−1^) containing ChETA or eYFP vectors was injected into the SN region (AP=−3.28, ML=−1.5, DV=4.3) in the ChETA+light and eYFP+light groups, respectively; no virus was injected into the Control group. Three mice in the ChETA+light group were used to quantitate the percentage of ChETA-positive cells that were also GFAP-positive: the ChETA-positive or GFAP-positive cells were counted and summed up from 10 tissue sections separately. The number of GFAP-positive cells was expressed as a percentage of the total ChETA-positive cells.

One week after virus injection or cell transplantation, we used a blue-light-emitting optical fibre with a diameter of 200 μm to illuminate the SN or striatal region with 100 ms pulses at intervals of 5 min for a total of 30 min in the eYFP+light and ChETA+light groups. The distance between the optical fibre tip and the cell implantation position was ~100μm (SN region: AP=−3.28, ML=−1.5, DV=4.2; or striatum region: AP=0.5, ML=−2.0, DV=4.2), and the average output power was ~10 mW mm^−2^. The light stimulation was conducted once a week and lasted for 3 weeks in eYFP+light and ChETA+light groups. To inhibit the FGFR, 20 μM FGFR inhibitor SU5402 (Tocris Bioscience, UK) was injected into the SN region before each light stimulation. One week after the last light stimulation, the mice were perfused with 4% PFA and the brain tissue was removed for bFGF quantification and immunostaining analysis.

### Microdialysis and determination of dopamine levels

The mice were anaesthetized with sodium pentobarbital (100 mg kg^−1^) and placed in a stereotaxic frame. A microdialysis probe (MER-10 mm guide, 2 mm membrane, Bioanalytical Systems) was implanted into the striatum region (AP=0.5, ML=−2.0, DV=4.3). The probes were perfused with artificial cerebrospinal fluid (NaCl 124 mM, KCl 3 mM, CaCl_2_ 2.4 mM, MgSO_4_ 1.3 mM, glucose 10 mM and HEPES 10 mM, pH=7.3) for 90 min before beginning the experiment, and samples were collected for 30 min in the experiment. The concentrations of dopamine in the dialysate were determined using a dopamine ELISA Kit (Abnova, Walnut, CA, USA), and the experiments were carried out according to the manufacturer’s instructions. In brief, dopamine in the dialysate is extracted using a cis-diol-specific gel, acylated and derivatized enzymatically. The optical density was determined at 450 nm using the microplate reader (Synergy 4, Biotek). The concentrations of dopamine were determined, normalized to that of the control group and compared among the different groups.

### Electrophysiology

Whole-cell recordings of astrocytes and DA neurons were performed using standard whole-cell patch-clamp techniques. For astrocytes, a Multiclamp 700B amplifier (Molecular Devices, Sunnyvale, CA, USA) was used to acquire cell recordings. The currents were low-pass filtered at 1KHz and digitized at 10KHz using a Digidata 1440A (Molecular Devices). A standard KCl pipette solution containing the following salts was used: 140 mM KCl, 4.3 mM MgCl_2_, 11 mM EGTA, 4.4 mM Na_2_ATP, and 10 mM HEPES sodium salt; the pH was adjusted to 7.2 using Tris base. Osmolarity was adjusted to 302 mOsm kg^−1^. Normal extracellular NaCl bath solution contained the following salts: 150 mM NaCl, 5 mM KCl, 3.31 mM CaCl_2_, 10 mM glucose and 10 mM HEPES acid. The pH was adjusted to 7.4 and the osmolarity was 306–312 mOsm kg^−1^. We used a DG-4 high-speed optical switch with a 300 W xenon lamp (Sutter Instruments, Novato, CA, USA) to deliver the light pulses to the ChETA-activated astrocytes. The output power was 3–5 Mw, and we used 10-Hz light stimulation (1–500 ms). The interval for the paired stimuli was 25–500 ms.We acquired the data using pClamp10software (Molecular Devices).

Recordings from DA neurons were identified by the action potential and the presence of a large inward current (Ih, >200 pA), evoked by a 1.5-s hyperpolarizing step from −50 to −150 mV. Whole-cell voltage-clamp recordings were made at a holding potential of −50 mV. The currents were filtered at 1 kHz, digitized at 2 kHz and collected on a personal computer using pClampsoftware. The micropipettes were made from borosilicate glass capillaries, with a resistance in the range of 3–6 MΩ. The pipette solutions used for whole-cell recordings contained 130 mMK-gluconate, 2 mM MgCl_2_, 10 mM HEPES, 1 mM EGTA, 3.5 mMMg-ATP, 1 mM Na-GTP and 10 mM Na-phosphocreatine (pH 7.3,280 mOsm kg^−1^). The artificial cerebrospinal fluid contained 126 mMNaCl, 2.5 mMKCl, 1.2 mM NaH_2_PO_4_, 1.2 mMMgCl_2_, 2.4 mM CaCl_2_, 11 mM glucose and 21.4 mM NaHCO_3_, saturated with 95%O_2_-5% CO_2_ (pH 7.4, 300 mOsm kg^−1^).

### Animal behaviour studies

For the grip strength test, forelimb grip strength was evaluated using a grip strength meter (Bioseb, France). The grip strength meter contains a digital force gauge attached to a grip strength platform having 3 mm size mesh. Mice in different groups were allowed to hold the platform with their forelimbs and pulled backwards with the tail in a horizontal plane. The maximum force applied to the platform immediately before the release of the paw grip was recorded in the meter. For the Pole Test, mice in different groups were placed vertically on a 30 cm vertical, 1 cm diameter pole. On the day before testing (day 1) the animals were habituated to the pole, and then the animals were recorded via digital video on the test day (day 2). The amounts of time were recorded for the mouse to turn towards the ground (time to orient down) and to reach the ground (time to descend). Rotational behaviour (0.5 mg kg^−1^ apomorphine, Sigma) was assessed at 4 weeks after the last light stimulation in a rotational system (Panlab, Spain), as previously described^53^. The results from the three different groups were expressed as contralateral net turns per 30 min, and the difference was compared among the three groups.The Open-Field test consisted of an 8-min session in the open-field chamber (50 × 50 cm), which was made of plastic and was divided into a central field (centre, 25 × 25 cm^2^) and a periphery field. Each individual mouse from the different groups was placed in the periphery field at the start of the test. Behaviours were recorded on video during the trial and the ANY-maze video tracking system (Stoelting, USA) was used for analysis.

### Statistical analysis

The experiment data were expressed as the mean±s.e.m. of the number of tests stated. Statistical comparisons were made using either Student’s *t*-test or analysis of variance (ANOVA) followed by Bonferroni’s multiple comparison’s *post-hoc* test, as indicated in the figure legends. All of the statistical tests were performed using the Statview (version 10.0, SPSS, Chicago, IL) program package or Prism 5.0 software. A *P* value <0.05 was taken as statistical significance.

## Author contributions

L.W. and F.Y. conceived and designed the experiments. Y.L., J.T., J.W., J.Z., B.W. and S.C. performed experiments. F.Y., Y.L. and J.T. analysed data. J.Z. and Y.M. helped to design the experiments and provided assistance.

## Additional information

**How to cite this article:** Yang, F. *et al*. Activated astrocytes enhance the dopaminergic differentiation of stem cells and promote brain repair through bFGF. *Nat. Commun.* 5:5627 doi: 10.1038/ncomms6627 (2014).

## Supplementary Material

Supplementary Information Supplementary Figures 1-9 and Supplementary Tables 1

Supplementary Movie 1 Rotational behavior in Control, eYFP+light and ChETA+light groups was assessed after the last light stimulation in a rotational system
