# Supplementary material for: Activated astrocytes enhance the dopaminergic differentiation of stem cells and promote brain repair through bFGF
Source: Nat Commun. 2014 Dec 17;5:5627. doi: 10.1038/ncomms6627 (PMC4284631; doi:10.1038/ncomms6627)
Supplement: Supplementary Information — Supplementary Figures 1-9 and Supplementary Tables 1 [file ncomms6627-s1.pdf]

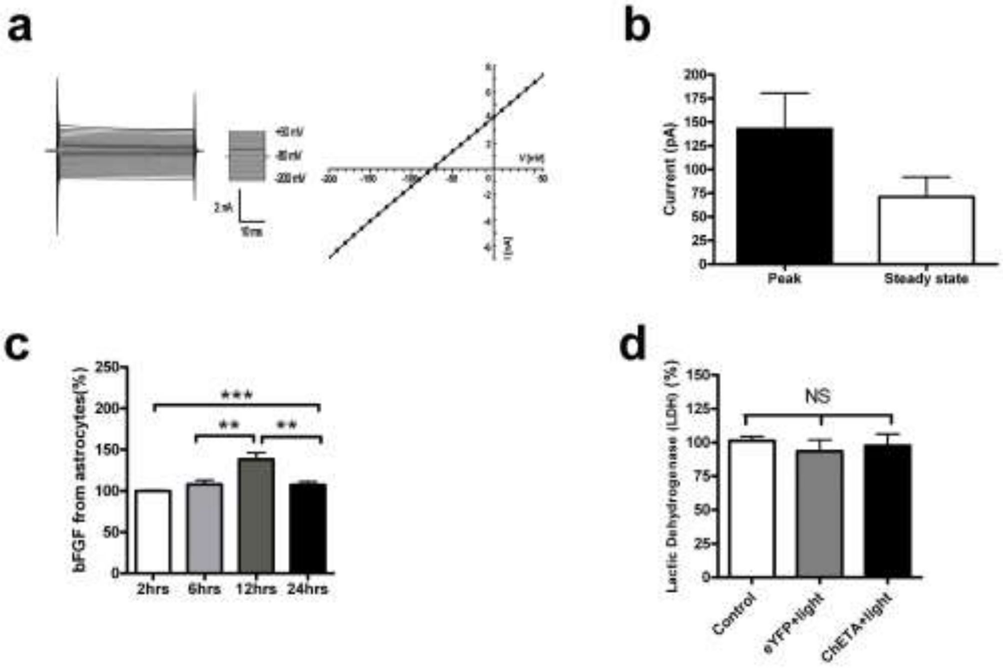

Supplementary Figure 1. Electrophysiological recordings from the astrocytes and quantification of bFGF and LDH

(a)IV curve of the cultured rat-midbrain-derived astrocytes.**(b)** The peak and steady-state amplitude of the induced depolarization current in astrocytes. (mean±s.e.m, n=6). **(c)** Quantification of released bFGF from astrocytes at 2, 6, 12 and 24 hours after the light stimulation. (mean±s.e.m, n=6). **(d)** Quantification of lactic dehydrogenase (LDH) release in the Control, eYFP+light and ChETA+light groups. (mean±s.e.m, n=6). All Student's t-test. **, p<0.01.***, p<0.001. NS, not significant.

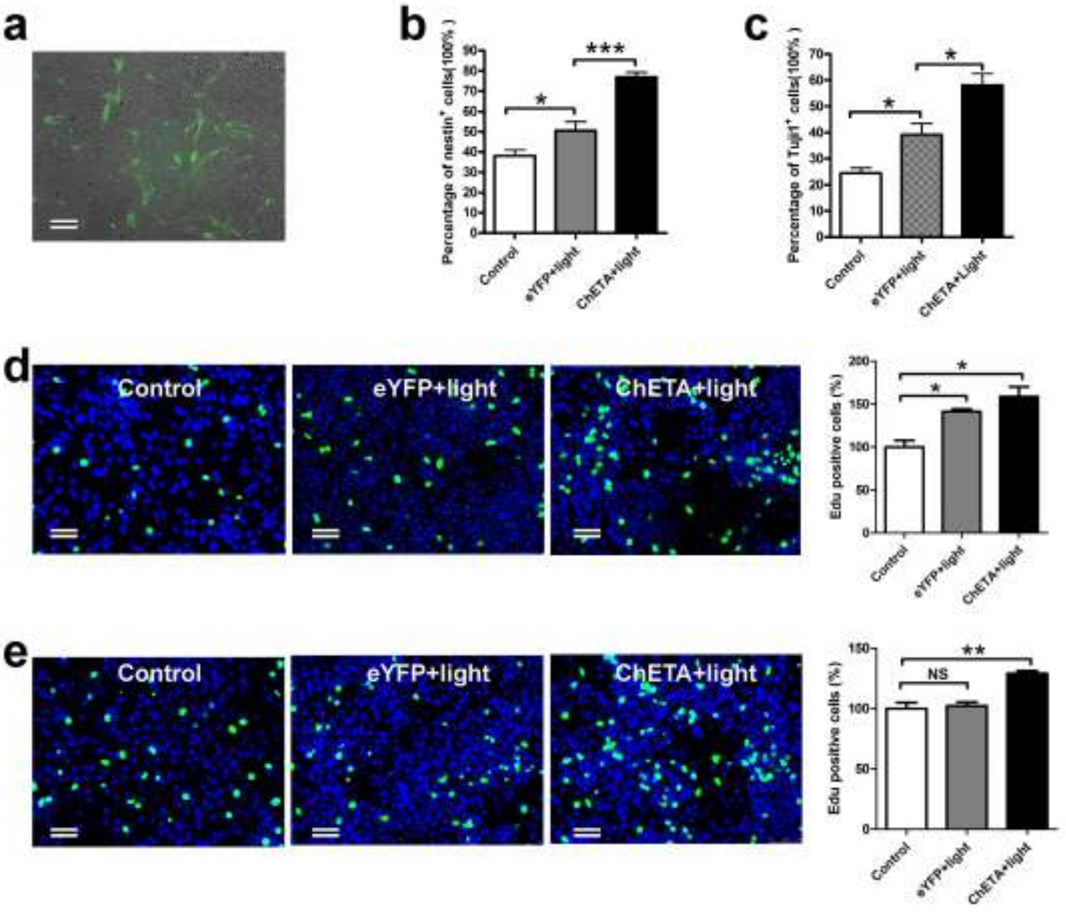

Supplementary Figure 2. Light stimulation enhanced the differentiation and proliferation of the stem cells

(a) ChETA-transfected astrocytes (green)were co-cultured with differentiated human embryonic stem cells, Bars=50μm.**(b)** The percentage of Nestin positive cells in the Control, eYFP+light and ChETA+light groups. (mean±s.e.m n=8). **(c)**The percentage of Tuj1 positive cells in the Control, eYFP+light and ChETA+light groups. (mean±s.e.m,n=8). **(d)**5-ethynyl-2-deoxyuridine (EdU)-incorporated cells were observed (left panel, green)and percentage of positive cells in the control, eYFP+light and ChETA+light groups (right panel) during the neural differentiation of the stem cells, (mean±s.e.m, n=10). **(e)**EDU-incorporated cells were observed (left panel, green)and the percentage of positive cells wasquantified in the control, eYFP+lightand ChETA+light groups during the dopaminergic differentiation of the stem cells. (mean±s.e.m, n=10) (right panel). All Student's t-test. *, p<0.05.**, p<0.01.***, p<0.001. NS, not significant.

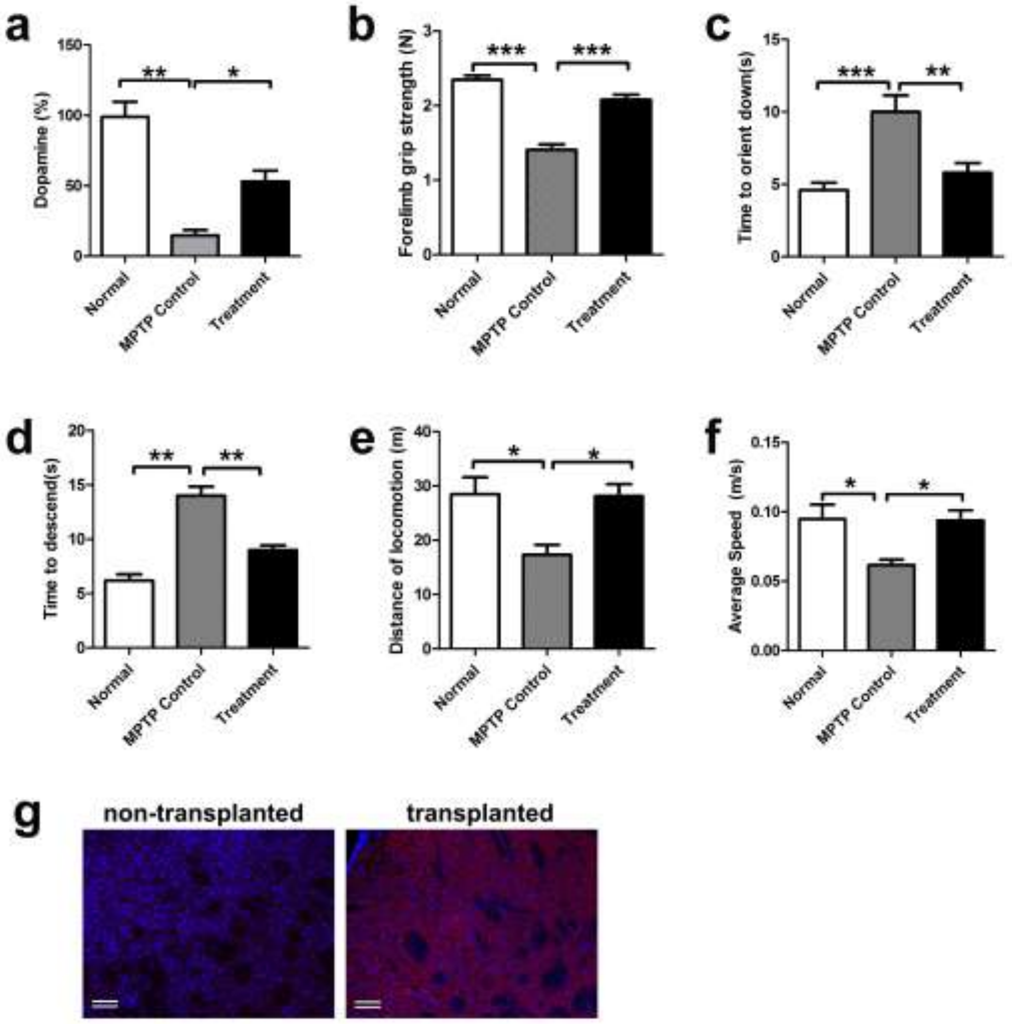

Supplementary Figure 3. Dopamine level and Motor function tests after transplanting the differentiated dopaminergic neurons into substantianigra

(a) The levels of dopamine in the dialysate from the striatum of mice in Normal, MPTP control and Treatment groups, (mean±s.e.m, n=3). **(b)** Forelimb grip strength in Normal, MPTP control and Treatment groups, (mean±s.e.m, n=5). **(c)** Time to orient down in the pole test for Normal, MPTP control and Treatment groups, (mean±s.e.m, n=5).**(d)** Time to descend in the pole test for Normal, MPTP control and Treatment groups, (mean±s.e.m, n=5). **(e)** Distance of locomotion in the open field test forNormal, MPTP control and Treatment groups, (mean±s.e.m, n=5). **(f)**Average speed in the open field test for Normal, MPTP control and Treatment groups, (mean±s.e.m, n=5). **(g)** TH staining in the transplanted side and non-transplanted side of the striatum . Bars=80μm. All Student's t-test.*, $p<0.05$.**, $p<0.01$.***, $p<0.001$.

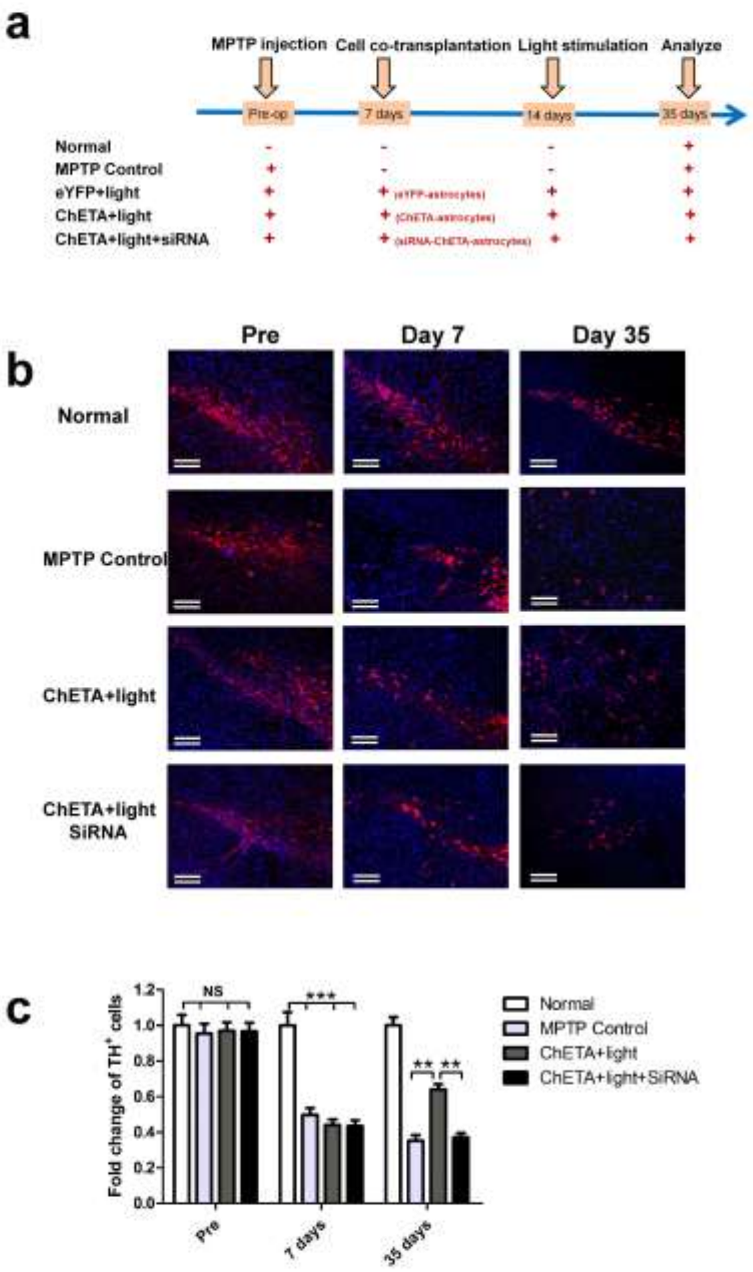

Supplementary Figure 4. Schematic flowchart for the co-transplantation experiment and change of endogenous TH neurons in different groups

(a) A schematic flowchart of the various manipulations in different groups in the co-transplantation experiment. **(b)**Immunostaining of endogenous TH-positive cells in Normal, MPTP Control, ChETA+light and ChETA+light+siRNA groups, Bars=50μm.**(c)**Fold change of TH^+ neurons at specific stages of MPTP degenerationand treatment. The number of TH^+ neurons in ChETA+light group was significantly higher than that of MPTP control group or siRNA group after the light stimulation, (mean±s.e.m, n=3). One-way ANOVA followed by Bonferroni's multiple comparison 's post-hoc test. **, p<0.01.***, p<0.001. NS, not significant.

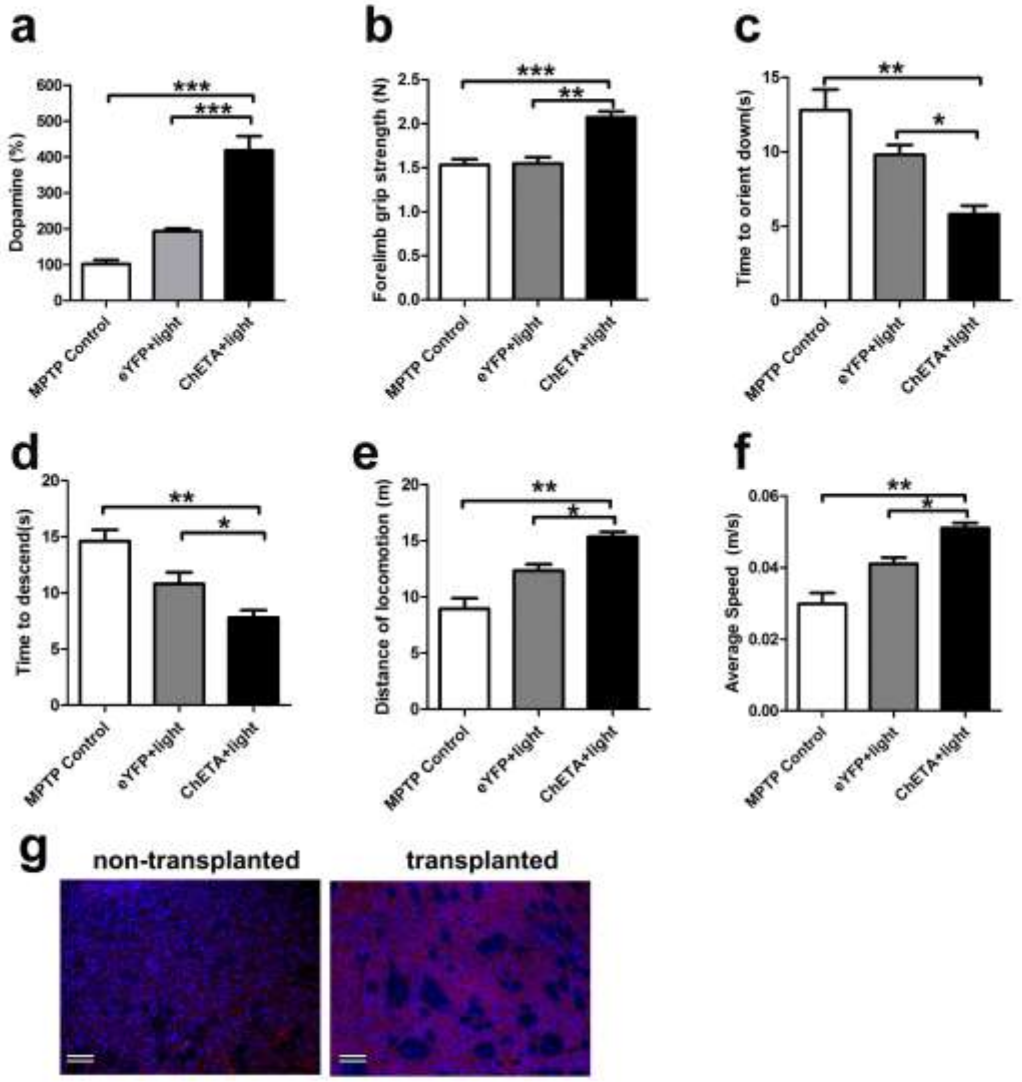

Supplementary Figure 5. Dopamine level and Motor function tests in the substantianigra co-transplantation experiment

(a)The levels of dopamine in the dialysate from the striatum of mice in MPTP control, eYFP+light and ChETA+light groups, (mean±s.e.m n=3). (b) Forelimb grip strength in MPTP control, eYFP+light and ChETA+light groups, (mean±s.e.m n=5). (c) Time to orient down in the pole test for MPTP control, eYFP+light and ChETA+light groups, (mean±s.e.m, n=5). (d) Time to descend in the pole test for MPTP control, eYFP+light and ChETA+light groups, (mean±s.e.m, n=5). (e) Distance of locomotion in the open field test for MPTP control, eYFP+light and ChETA+light groups, (mean±s.e.m, n=5). (f) Average speed in the open field test for MPTP control, eYFP+light and ChETA+light groups, (mean±s.e.m, n=5). (g) TH staining in the transplanted side and non-transplanted side of the striatum. Bars=80μm. All Student's t-test. *, p<0.05.**, p<0.01.***, p<0.001.

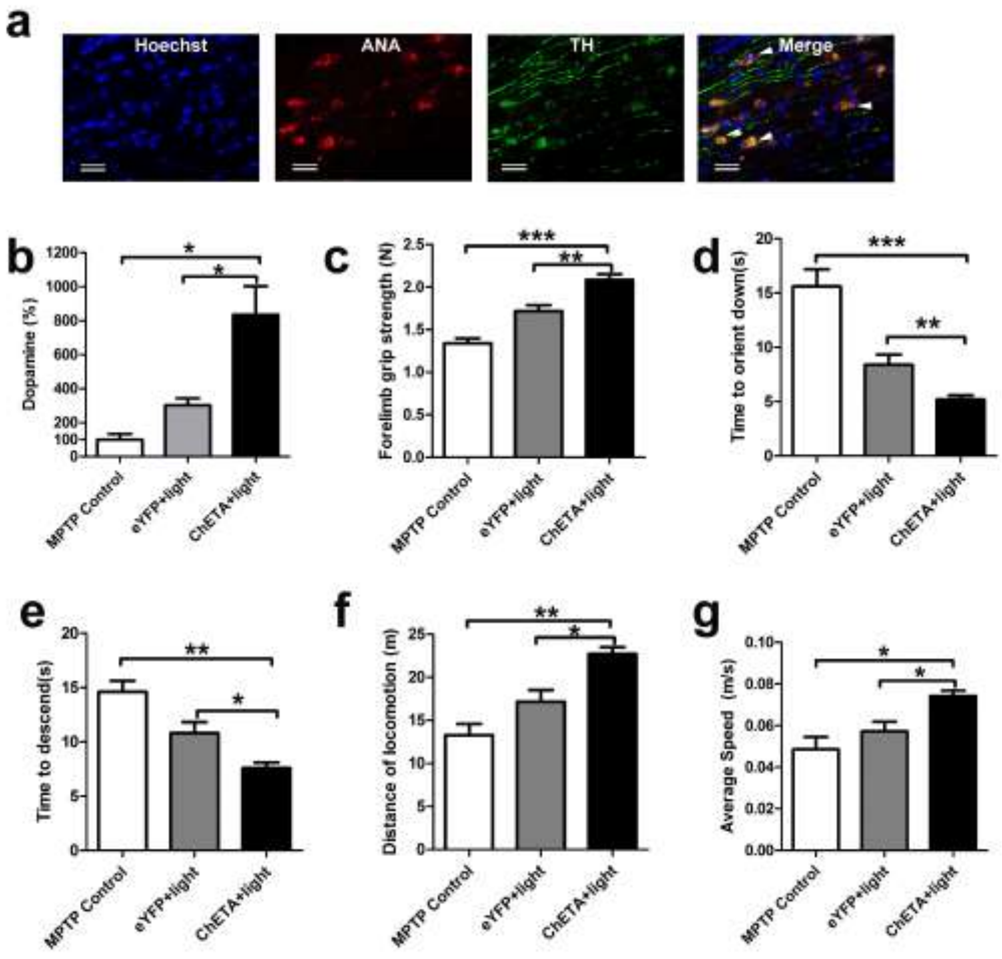

Supplementary Figure 6. Light stimulationpromotesbrain repair after co-transplanting the astrocytes and ES-derived stem cells into the striatum in MPTP model

(a) Immunostaining of TH (green) and ANA (red) of the transplanted stem cells in striatum region. Bars=20μm. **(b)**The levels of dopamine in the dialysates from the striatum of mice in MPTP control, eYFP+light and ChETA+light groups, (mean±s.e.m, n=3).**(c)** Forelimb grip strength in MPTP control, eYFP+light and ChETA+light groups, (mean±s.e.m, n=5).**(d)** Time to orient down in the pole test for MPTP control, eYFP+light and ChETA+light groups, (mean±s.e.m n=5). **(e)** Time to descend in the pole test for MPTP control, eYFP+light and ChETA+light groups, (mean±s.e.m, n=5). **(f)** Distance of locomotion in the open field test for MPTP control, eYFP+light and ChETA+light, (mean±s.e.m, n=5). **(g)** Average speed in the open field test for MPTP control, eYFP+light and ChETA+light groups, (mean±s.e.m, n=5). All Student's t-test. *, $p<0.05$.**, $p<0.01$.***, $p<0.001$.

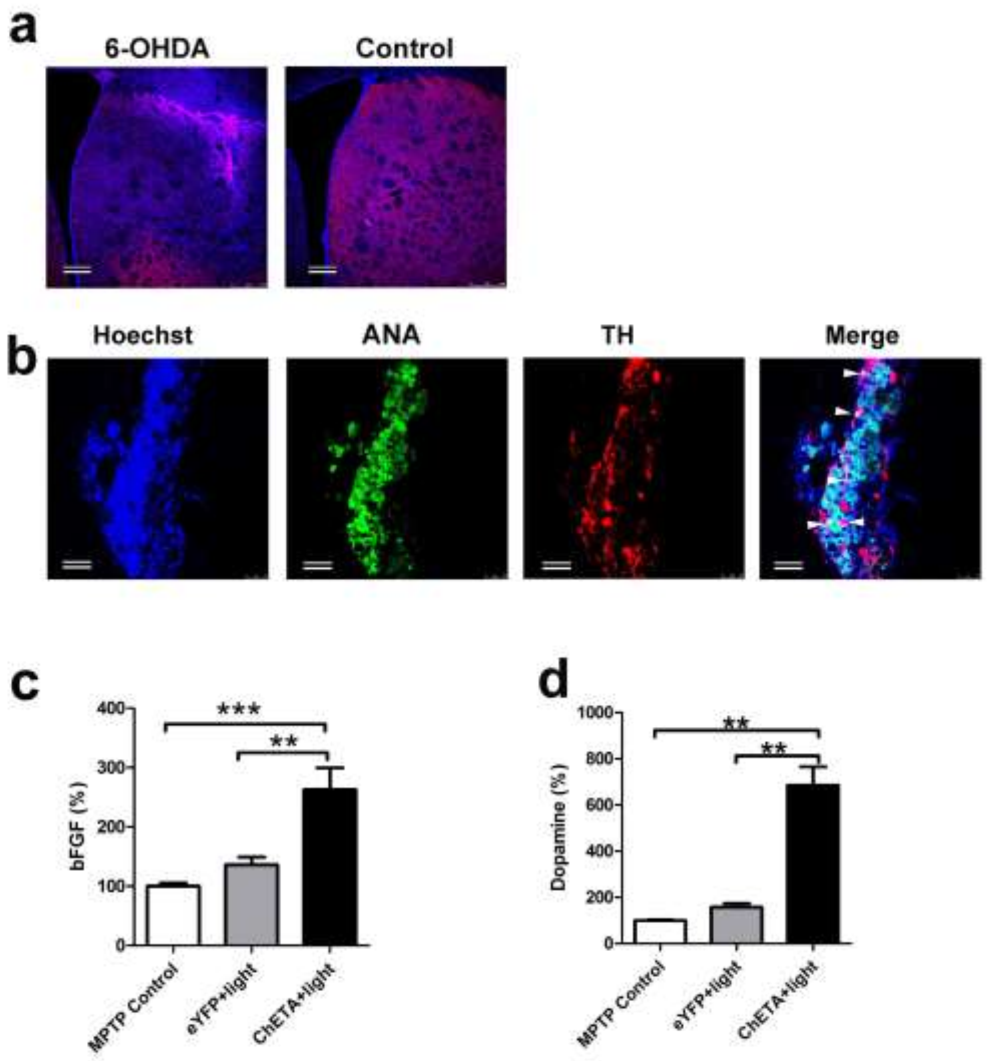

Supplementary Figure 7. Light stimulation induced dopaminergic differentiation of stem cells, and increasedbFGF and dopamine level after striatal transplantationin 6-OHDAmodel

(a) Immunostaining of TH in striatum in 6-OHDA-induced PD model and control. Bars=150μm**(b)**Immunostaining of TH (green) and ANA (red) of the transplanted stem cells in striatum region. Bars=40μm. **(c)**Quantification of bFGF from striatum tissue in the Control, eYFP+light and ChETA+light groups. (mean±s.e.m, n=3).**(d)**The levels of dopamine in the dialysate from the striatum of mice in MPTP control, eYFP+light and ChETA+light groups, (mean±s.e.m, n=3). All Student's t-test. **, *p*<0.01.***, *p*<0.001

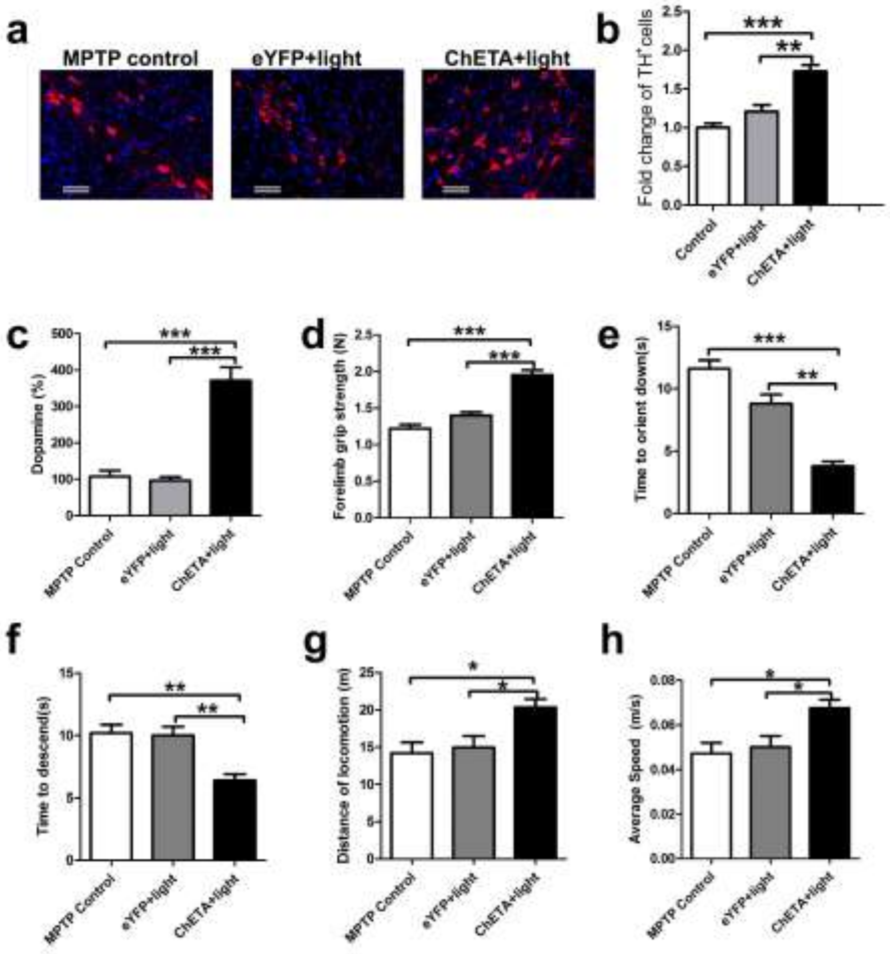

Supplementary Figure 8. Light stimulation of endogenous astrocytespromotesfunctional repair and amelioration of the behavior deficits in the MPTP model

(a) Immunostaining of endogenous TH-positive cells in MPTP Control, eYFP+light and ChETA+light groups. Bars=50μm. **(b)**Fold change of TH^+ neurons in the three different groups, (mean±s.e.m, n=3). **(c)**The levels of dopamine in the dialysate from the striatum of mice in MPTP control, eYFP+light and ChETA+light groups, (mean±s.e.m, n=3).**(d)** Forelimb grip strength in MPTP control, eYFP+light and ChETA+light groups, (mean±s.e.m, n=5). **(e)** Time to orient down in the pole test for MPTP control, eYFP+light and ChETA+light groups, (mean±s.e.m, n=5).**(f)** Time to descend in the pole test for MPTP control, eYFP+light and ChETA+light groups, (mean±s.e.m, n=5). **(g)** Distance of locomotion in the open field test for MPTP control, eYFP+light and ChETA+light groups, (mean±s.e.m, n=5). **(h)** Average speed in the open field test for MPTP control, eYFP+light and ChETA+light groups, (mean±s.e.m, n=5). All Student's t-test. *, $p<0.05$.**, $p<0.01$.***, $p<0.001$.

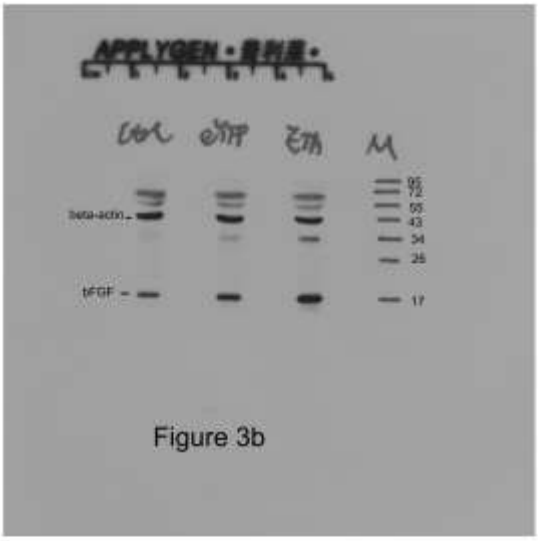

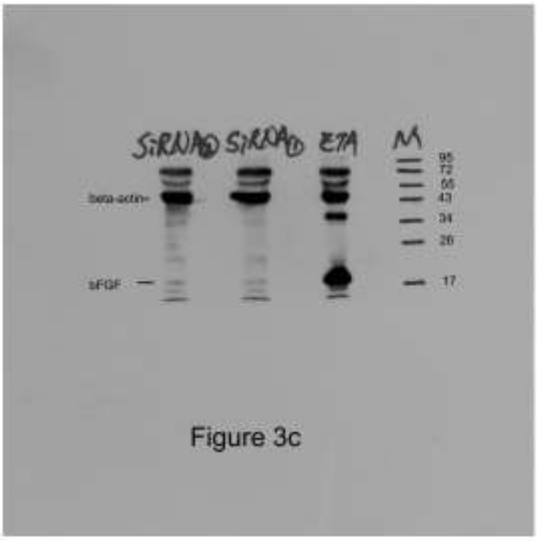

Supplementary Figure 9. Original scans of western blots shown in main text

(left) original scan of western blot shown in Figure 3b.
(right) original scan of western blot shown in Figure 3c.

Supplementary Table 1

Gene	Primer sequence	Tm(℃)	Product size
Gapdh	F 5'-ACCACAGTCCATGCCATCAC-3'<br>R 5'-TCCACCACCCTGTTGCTGTA-3'	57	452bp
Tuj1	F 5'-GTTCCCACGTCTCCACTTCTTC-3'<br>R 5'-CCAGGTCATTCATGTTGCTCTC-3'	55	479bp
TH	F 5'-GGTTCCCAAGAAAAGTGTCAG-3'<br>R 5'-GGTGTAGACCTCCTTCCAG-3'	55	218bp
Nurr	F 5'-CGGACAGCAGTCCTCCATTAAGGT-3'<br>R 5'-CTGAAATCGGCAGTACTGACAGCG-3'	61	712bp
DAT	F 5'-AGCAGAACGGAGTGCAGCT-3'<br>R 5'-GTATGCTCTGATGCCGTCT-3'	55	785bp
